# Nanoscaled biphasic calcium phosphate modulates osteogenesis and attenuates LPS-induced inflammation

**DOI:** 10.3389/fbioe.2023.1236429

**Published:** 2023-11-29

**Authors:** Yi-Chun Su, Trinh T. T. Phan, Tzu-Wei Wang, Shao-Hsuan Chang, Feng-Huei Lin, Tzu-Sheng Hsu, Lih-Yuan Lin

**Affiliations:** ^1^ Institute of Molecular and Cellular Biology, College of Life Sciences and Medicine, National Tsing Hua University, Hsinchu, Taiwan; ^2^ Department of Materials Science and Engineering, National Tsing Hua University, Hsinchu, Taiwan; ^3^ School of Engineering, University of Liverpool, Liverpool, United Kingdom; ^4^ Professional Master’s Program of Biotechnology Management, National Taiwan University, Taipei, Taiwan; ^5^ Institute of Biomedical Engineering, National Taiwan University, Taipei, Taiwan; ^6^ Institute of Biomedical Engineering and Nanomedicine, National Health Research Institutes, Miaoli, Taiwan

**Keywords:** nanoscaled biphasic calcium phosphate nanomaterials, biphasic materials, macrophages, osteoblasts, inflammatory response, immunomodulation, anti-inflammation

## Abstract

Micron-scale structure biphasic calcium phosphate (BCP) materials have demonstrated promising clinical outcomes in the field of bone tissue repair. However, research on biphasic calcium phosphate materials at the nanoscale level remains limited. In this study, we synthesize granular-shaped biphasic calcium phosphate nanomaterials with multiple desirable characteristics, including negatively charged surfaces, non-cytotoxicity, and the capability to penetrate cells, using a nanogrinding dispersion process with a polymeric carboxylic acid as the dispersant. Our results reveal that treating human osteoblasts with 0.5 μg/mL biphasic calcium phosphate nanomaterials results in a marked increase in alkaline phosphatase (ALP) activity and the upregulation of osteogenesis-related genes. Furthermore, these biphasic calcium phosphate nanomaterials exhibit immunomodulatory properties. Treatment of THP-1-derived macrophages with BCP nanomaterials decreases the expression of various inflammatory genes. Biphasic calcium phosphate nanomaterials also mitigate the elevated inflammatory gene expression and protein production triggered by lipopolysaccharide (LPS) exposure in THP-1-derived macrophages. Notably, we observe that biphasic calcium phosphate nanomaterials have the capacity to reverse the detrimental effects of LPS-stimulated macrophage-conditioned medium on osteoblastic activity and mineralization. These findings underscore the potential utility of biphasic calcium phosphate nanomaterials in clinical settings for the repair and regeneration of bone tissue. In conclusion, this study highlights the material properties and positive effects of biphasic calcium phosphate nanomaterials on osteogenesis and immune regulation, opening a promising avenue for further research on inflammatory osteolysis in patients undergoing clinical surgery.

## 1 Introduction

Osteoarthritis (OA) is a prevalent degenerative joint disease affecting millions of people worldwide and represents the most common form of arthritis. Total joint replacement (TJR) surgery is a widely used procedure for the treatment of advanced OA. However, osteolysis presents a significant challenge associated with TJR surgery and can lead to prosthesis failure and costly revision surgery, placing a substantial economic burden on both patients and healthcare systems (Gallo et al., 2013). Inflammatory osteolysis arises from various factors, including the dysregulation of osteoclast formation and function triggered by inflammatory cytokines (Mbalaviele et al., 2017). Gram-negative bacterial infections, which enhance osteoclastic activity and osteolysis, can lead to complications such as osteomyelitis and infections of orthopedic implants (Hara et al., 1998). Lipopolysaccharide (LPS), a key component of the outer cell membrane of Gram-negative bacteria, is a major mediator of inflammation and osteolysis. LPS induces the production of pro-inflammatory cytokines, initiating an inflammatory response (Poltorak et al., 1998; Qureshi et al., 1999). Macrophages, particularly those with a pro-inflammatory phenotype, play a critical role in the development of this condition by secreting various pro-inflammatory cytokines, including interleukin (IL)-6, IL-1β, and tumor necrosis factor-alpha (TNF-α). These cytokines increase osteoclast activity while inhibiting the osteogenic process, contributing to the progression of inflammatory osteolysis (Amarasekara et al., 2018). Thus, controlling inflammation and preventing complications are crucial aspects of optimal bone healing and regeneration.

In recent years, biomaterials based on nanoparticles have gained significant interest because of their unique physical, chemical, and biological properties. Nanoparticles have distinct characteristics, including significantly increased surface area and enhanced cellular absorption rates. Their high surface area-to-volume ratio renders them highly reactive, leading to increased biological activity (Salata, 2004; Xie et al., 2010; Remya et al., 2014). In the field of bone tissue engineering, nanoparticles have been used to enhance the mechanical and biological properties of scaffolds used for bone regeneration. They also aid in the prevention of inflammatory osteolysis (Tautzenberger et al., 2012; Whitaker et al., 2021; Zheng et al., 2021). Hydroxyapatite (HAP) and tricalcium phosphate (TCP) are commonly used biomaterials in bone tissue engineering. HAP, a naturally occurring form of calcium phosphate, constitutes the largest amount of inorganic components in human bones. While HAP is known to be osteoconductive its osteoinductive properties are relatively weak (Ogata et al., 2005; Yoshikawa and Myoui, 2005; Tsiridis et al., 2006; Samavedi et al., 2013; Awasthi et al., 2020). HAP coatings applied onto metallic implants enhance the activity of osteoblasts and augment bone-implant contact areas, resulting in superior biological fixation, biocompatibility, and bioactivity for clinical applications (Morris and Ochi, 1998; Darimont et al., 2002; Ramires et al., 2003). TCP (Ca_3_(PO4)_2_) has a higher solubility and resorption rate than HAP and is widely used to increase biocompatibility (Yamada et al., 1997; Horch et al., 2006). In addition, TCP stimulates the proliferation of osteoprecursor cells, including osteoblasts and bone marrow stromal cells (Yao et al., 2004). Thus, BCP materials combining HAP and TCP properties have been developed to augment bone tissue regeneration. This combination increases bioactivity, bioresorbability, and osteoinductivity, making it an effective choice for bone grafting and bone substitute materials (Arinzeh et al., 2005; Amirian et al., 2015; He et al., 2016). While previous studies have primarily focused on nano-HAP applications in osteogenesis regulation (Yang et al., 2018), this study represents the first attempt to manufacture BCP nanoparticles through nanogrinding and to explore the effects of BCP nanoparticles on osteogenesis and immune regulation. Moreover, the characteristics of the nano-level BCP material, including its cytotoxicity, effects on osteogenesis, and immunomodulation have been evaluated in this study.

Previous studies have presented various methods for preparing calcium phosphate nanoparticles, including precipitation, sol-gel, hydrothermal processing, flame spray pyrolysis, and microemulsion-based methods (Liu et al., 2002; Lin et al., 2009; Teoh et al., 2010; Richard et al., 2017; Yang and Park, 2019). However, the commonly used precipitation method preparations often result in the formation of needle-like particles. Despite the cost-efficiency of the precipitation method, the needle-like morphology raises concerns because needle-like particles exhibit lower cell uptake efficiency and may lead to cytotoxicity when compared to granular-shaped particles (Egbuna et al., 2021). Furthermore, needle-like particles tend to agglomerate or bundle together, affecting their dispersion in polymeric matrices or biological fluids. Such issues impair cellular uptake and increase toxicity, thereby diminishing biomaterial compatibility (Zhang et al., 2017; Wang et al., 2019; Egbuna et al., 2021). Additionally, it is crucial to incorporate an appropriate dispersant to prevent nanoparticle aggregation during the distribution of nanoparticles, as various dispersants have been employed in the nanoparticle preparation process (Iijima et al., 2009; Hartmann et al., 2015). Thus, alternative efficient methods to ensure BCP material compatibility need to be defined. In this study, we used a nanogrinding method with a polymeric carboxylic acid as the dispersant to produce granular-shaped nanoparticles, which are expected to exhibit better cellular uptake and reduced cytotoxicity compared with needle-like particles.

Our study aimed to develop an orthopedic biomaterial with enhanced osteogenesis and immunomodulatory capabilities that reduces pro-inflammatory factors in the local immune microenvironment to promote osteogenesis. Through *in vitro* evaluations of the synthesized BCP nanoparticles, including assessments of cytotoxicity, effects on osteogenesis, and immunomodulatory activities, we found that these nanoparticles presented good biocompatibility, promoted osteogenesis, and modulated the immune response by diminishing the expression of pro-inflammatory cytokines in THP-1-derived macrophages. Our results highlight that granular-shaped BCP nanoparticles prepared by nanogrinding may have significant potential as an effective biomaterial for bone tissue engineering and for preventing inflammatory osteolysis in patients undergoing total joint replacement surgery.

## 2 Materials and methods

### 2.1 Chemical reagents and drugs

Reagents for cell culture were purchased from Invitrogen Gibco (Grand Island, NY, USA). Chemicals were purchased from Sigma-Aldrich (St. Louis, MO, USA) unless otherwise specified. WST-1 used for the cytotoxicity test was purchased from Takara Bio Inc. (Shiga, Japan). Primers for qRT-PCR were purchased from Integrated DNA Technologies (Coralville, IA, USA).

### 2.2 Preparation of nanoparticles

Micro-level hydroxyapatite (HAP)/β-tricalcium phosphate (β-TCP) granules (60%/40%) with a size between 250 and 500 μm were obtained from Wiltrom Co., Ltd. (Hsinchu, Taiwan). To prepare nano-HAP/β-TCP, a wet polish method was employed using a nanobead mill (JBM-B035; Just Nanotech, Tainan, Taiwan). Micro-HAP/β-TCP granules were mixed with a polymeric carboxylic acid as the dispersant (OROTAN™ 1850E Dispersant at a concentration of 1%) and grounded using a 100 μm zirconia balls at 2400 rpm for 1 h. Following centrifugation to remove the zirconia balls, the supernatant was collected, filtered using a 0.22 μm filter membrane, and stored at room temperature before use in different assays.

### 2.3 Characterization of nanoparticles

Nano-HAP/β-TCP morphology was analyzed using transmission electron microscopy (TEM; JEOL JEM-F200, Tokyo, Japan) with an accelerating voltage of 200 keV. The crystal structure of nano-HAP was analyzed using energy-dispersive X-ray diffractometry (Bruker D2 PHASER, Billerica, Massachusetts, USA) with Cu-Kα radiation. Zeta potential measurements were conducted in distilled water to determine the particle size and surface charge using a Malvern Zetasizer 3000 HS instrument (Malvern Instruments, Worcestershire, UK).

### 2.4 Cell culture procedure

#### 2.4.1 hFOB 1.19 cell line

The human fetal osteoblastic cell line hFOB 1.19 (purchased from the Bioresource Collection and Research Center (BCRC), Hsinchu, Taiwan) was cultured in a 1:1 mixture of Ham’s F12 medium and Dulbecco’s Modified Eagle’s Medium (DMEM), supplemented with 10% heat-inactivated fetal bovine serum (FBS), 2 mM L-glutamine, 0.3 mg/mL G418, and 100 units/mL penicillin/streptomycin. Cells were cultured at 34°C in a 5% CO_2_ humidified atmosphere. To induce osteogenesis, hFOB 1.19 cells were cultured in an osteogenic differentiation medium containing 10^−8^ M dexamethasone, 10 mM β-glycerol phosphate, and 50 mM ascorbate-2-phosphate at 39.5°C in a 5% CO_2_ humidified atmosphere.

#### 2.4.2 THP-1-derived macrophages

The THP-1 cell line, derived from acute monocytic leukemia, was maintained in Roswell Park Memorial Institute (RPMI) 1640 medium supplemented with 10% FBS, 0.22% sodium bicarbonate, 2 mM L-glutamine, and 100 units/mL penicillin/streptomycin. Cells were cultured at 37°C in 5% CO_2_. Differentiation of THP-1 cells (2 × 10^6^ cells/well in 6-well plates) into macrophage-like cells was achieved by 50 nM phorbol 12-myristate 13-acetate (PMA) treatment for 48 h.

### 2.5 Cell viability and cell proliferation assay

Cell viability in response to nanoparticle treatments was assessed by measuring cellular dehydrogenase activity using the WST-1 assay (Takara Bio Inc., Shiga, Japan). Cells were seeded in 96-well cell microplates at a density of 3 × 10^4^ cells/well for hFOB 1.19 cells or 5 × 10^4^ cells/well for THP-1-derived macrophages in 200 µL of complete growth medium. After 1 day, the medium was renewed and cells were exposed to the indicated concentration of nanoparticles (0.5, 5, or 50 μg/mL). The WST-1 assay was performed according to the manufacturer’s instructions. The absorbance was measured at a wavelength of 450 nm using an iMark™ Microplate Reader (BIO-RAD, Hercules, CA, USA).

Cell proliferation assays were performed on hFOB 1.19 cells seeded in 6-well plates at a density of 2 × 10^4^ cells/well in 2 mL of complete growth medium. After 24 h, the medium was changed and the cells were exposed to the indicated nanoparticle concentration (0.5, 5, or 50 μg/mL). Cell counts were measured at different incubation time points using a hemocytometer.

### 2.6 Measurement of ROS

Intracellular reactive oxygen species (ROS) levels were detected using the 2′,7′-dichlorofluorescein (DCFH-DA) assay. Cells were seeded in 24-well plates at a density of 1.2 × 10^5^ cells/well for hFOB 1.19 cells or 2 × 10^6^ cells/well for THP-1-derived macrophages. After 24 h, cells were treated with various concentrations of nano-HAP/β-TCP particles for 48 h. To detect intracellular ROS levels, 5 μM DCFH-DA was added, and the cells were incubated for 30 min at 37°C. The DCF fluorescence intensity produced following oxidation (525 nm) was measured by flow cytometry as an indicator of oxidative stress using a BD Accuri™ C6 flow cytometer (BD Biosciences, San Jose, CA, USA).

### 2.7 Nanoparticle absorption assay (TEM)

The cellular uptake of nano-HAP/β-TCP by hFOB 1.19 cells and THP-1-derived macrophages was visualized by transmission electron microscopy (TEM) (FEI Tecnai™ G2 F-20 S-TWIN, Hillsboro, Oregon, USA). Cells were seeded at a density of 2 × 10^5^ cells/well on a plastic slide and cultured for 24 h. Nano-HAP/β-TCP particles (0.5 μg/mL or 50 μg/mL for hFOB 1.19 cells and 50 μg/mL for THP-1-derived macrophages) were then added to the medium and incubated for an additional 24 h. The cells were fixed with a pre-fixative solution containing 2.5% glutaraldehyde and 1% tannic acid in 1x PBS at 4°C for 1 h. The samples were then treated with a 1% OsO_4_ post-fixative solution (#19190; Electron Microscopy Sciences, Hatfield, PA, USA) in 1x PBS for 1 h and then incubated in 1% uranyl acetate (#22405, Electron Microscopy Sciences) for 30 min. After dehydration with ethanol, the cells were permeabilized and embedded in epoxy resin. Ultrathin sections were counterstained with 4% uranyl acetate and lead citrate and examined by transmission electron microscopy.

### 2.8 Osteogenesis study

#### 2.8.1 ALP activity assay

hFOB 1.19 cells were seeded at a density of 2×10^4^ cells/well in 24-well plates containing 1 mL of complete growth medium. Cells were treated with various concentrations of nanoparticles (0.5, 5, and 50 μg/mL) in fresh medium, and ALP activity was assessed at different time points (days 0, 1, 3, 5, and 7). Briefly, cells were washed twice with PBS and lysed with 200 μL of lysis buffer (25 mM Tris-HCl and 0.5% Triton X-100) at 4°C for 2 h. After complete lysis, 50 μL of lysate was added to a 96-well plate, followed by the addition of 50 mmol/L p-nitrophenyl phosphate (p-NPP) in sodium carbonate buffer at pH = 10.4. The mixture was then incubated at 37°C for 30 min. The amount of p-NPP released was measured using an iMark™ Microplate Reader (BIO-RAD, Hercules, CA, USA). The level of ALP activity in the control cell cultures was considered to be 100%.

#### 2.8.2 qRT-PCR

Total RNA was extracted from cells using TRIzol reagent (Invitrogen, Carlsbad, CA, USA) according to the manufacturer’s instructions, and cDNA was generated using the RevertAid First Strand cDNA Synthesis Kit (Invitrogen, Carlsbad, CA, USA). Gene expression levels were measured using quantitative real-time PCR (qRT-PCR) with SYBR Green PCR Master Mix (Cat. No. 4309155; Applied Biosystems, Foster City, CA, USA) on a StepOnePlus Real-Time PCR system (Applied Biosystems, Foster City, CA, USA). Data were acquired and analyzed using StepOne Software v2.3 (Thermo Fisher Scientific, Leicestershire, UK). The expression of glyceraldehyde 3-phosphate dehydrogenase (GAPDH) in hFOB 1.19 cells or ubiquitin C (UBC) in THP-1-derived macrophages was determined in each sample and used as a reference gene. The expression of target genes was compared using the 2^−ΔΔCT^ method (Livak and Schmittgen, 2001). The primer sequences used are shown in Table 1.

**TABLE 1:** Primer sequences used in this study.

Gene	Primer sequence (5′-3′)
*ALP*	Forward: CCACGTCTTCACATTTGGTG Reverse: GCAGTGAAGGGCTTCTTGTC
*Runx2*	Forward: CGCATTCCTCATCCCAGTAT Reverse: GACTGGCGGGGTGTAAGTAA
*OPN*	Forward: CCCACAGACCCTTCCAAGTA Reverse: ACACTATCACCTCGGCCATC
*IL-6*	Forward: AGACAGCCACTCACCTCTTCAG Reverse: TTCTGCCAGTGCCTCTTTGCTG
*IL-1β*	Forward: CTCGCCAGTGAAATGATGGCT Reverse: GTCGGAGATTCGTAGCTGGAT
*TNF-α*	Forward: TTCTGCCTGCTGCACTTTGGA Reverse: TTGATGGCAGAGAGGAGGTTG
*UBC*	Forward: CTGGAAGATGGTCGTACCCTG Reverse: GGTCTTGCCAGTGAGTGTCT
*GAPDH*	Forward: TGCACCACCAACTGCTTAGC Reverse: GGCATGGACTGTGGTCATGAG

### 2.9 LPS stimulation and ELISA

Lipopolysaccharides (LPS) from *Escherichia coli* (Sigma) were solubilized in sterile water. Macrophages derived from THP-1 cells were pretreated with LPS at a concentration of 20 ng/mL for 3 h. Subsequently, nano-HAP/β-TCP particles at the concentration of 0.5, 5, or 50 μg/mL were added to the cells and incubated for an additional 24 h. The protein concentrations of IL-1β, IL-6, and TNF-α in the culture supernatants were measured after a 24-h incubation period. This was accomplished using ELISA kits (Invitrogen, Carlsbad, CA, USA) in accordance with the manufacturer’s instructions.

### 2.10 Alizarin Red S staining

Quantification of extracellular matrix mineralization in cultured osteoblasts was performed using the Alizarin Red S (ARS, Sigma) staining technique, which detects bone nodules where the calcium was precipitated. Cells stained with ARS were observed under a light microscope (Nikon, Tokyo, Japan) connected to a Dino-Eye AM423X Digital Microscope Eyepiece Camera (AnMo Electronics Corporation, New Taipei City, Taiwan). The ARS dye that was incorporated into the cell cultures was extracted using a solution of 10% cetyl pyridinium chloride in 10 mM sodium phosphate (pH = 7.0) for 1 h at 37°C. The extracted stain was subsequently transferred to a 96-well plate, and the absorbance at 562 nm was quantified using an iMark™ Microplate Reader (BIO-RAD). The ARS stain level obtained from the control cell cultures was designated as the baseline, representing 100%. The amounts of calcium deposition in the various cell cultures subjected to different treatments were quantified and represented as a percentage relative to the control group.

### 2.11 Statistical analysis

Data were presented as mean ± standard deviation (SD). Statistical significance was determined by two-way analysis of variance (ANOVA) followed by Tukey’s test. Statistical significance was considered as *p < 0.05 and **p < 0.01.

## 3 Results

### 3.1 Preparation and characterization of BCP nanoparticles (nano-HAP/β-TCP)

**FIGURE 1:**
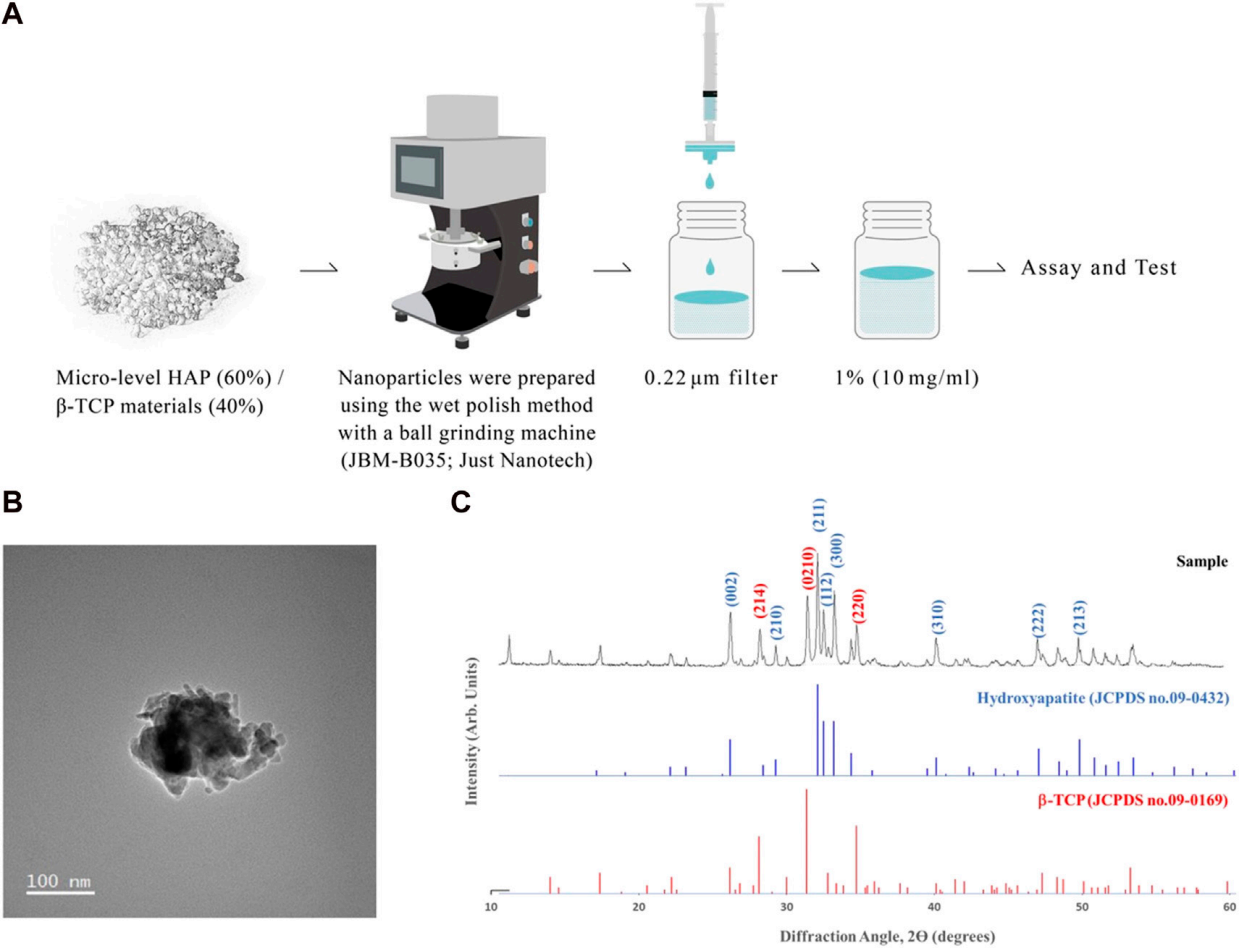
Characterization of nano-HAP/β-TCP particles. **(A)** Schematic depicting the preparation of nano-HAP/β-TCP particles. **(B)** Transmission electron microscopy (TEM) images and **(C)** X-ray diffractograms of the nano-HAP/β-TCP particles. Scale bar: 100 nm.

To synthesize the nano-level of HAP/β-TCP complex, we used the micro-level of HAP/β-TCP complex (250–500 μm) from Wiltrom Co., Ltd. with a HAP:TCP ratio of 60:40. Nano-HAP/β-TCP particles were prepared via the nanogrinding method. During the grinding process, the HAP/β-TCP complex was mixed with the dispersant to ensure the proper dispersion of the particles into the nanoscale, yielding a granular-shaped morphology (Figure 1A). Following the filtration process, the morphology and crystal structure of nano HAP/β-TCP particles were examined by transmission electron microscopy (TEM) and X-ray diffractometry (Figures 1B, C). TEM micrographs showed that the nano HAP/β-TCP particles were granular-shaped. The size of the nano HAP/β-TCP was determined to be 116.7 ± 1.438 nm. These particles exhibited a negative zeta potential of −51.35 ± 8.883 mV (Table 2). Scanning electron microscopy (SEM) analysis, along with Energy Dispersive X-ray (EDX) data, indicated a Ca/P ratio of 1.6 (Supplementary Figures S1A–C).

**TABLE 2:** Physical test of nano-HAP/β-TCP particles.

	Particle size (nm)	Zeta potential (mV)	HAP/β-TCP (w/w)
Biphasic nanomaterial	116.7 ± 1.438	−51.35 ± 8.883	56.2 ± 0.04/43.8 ± 0.04

### 3.2 Bio-reactivity of nano-HAP/β-TCP on human osteoblast cells

**FIGURE 2:**
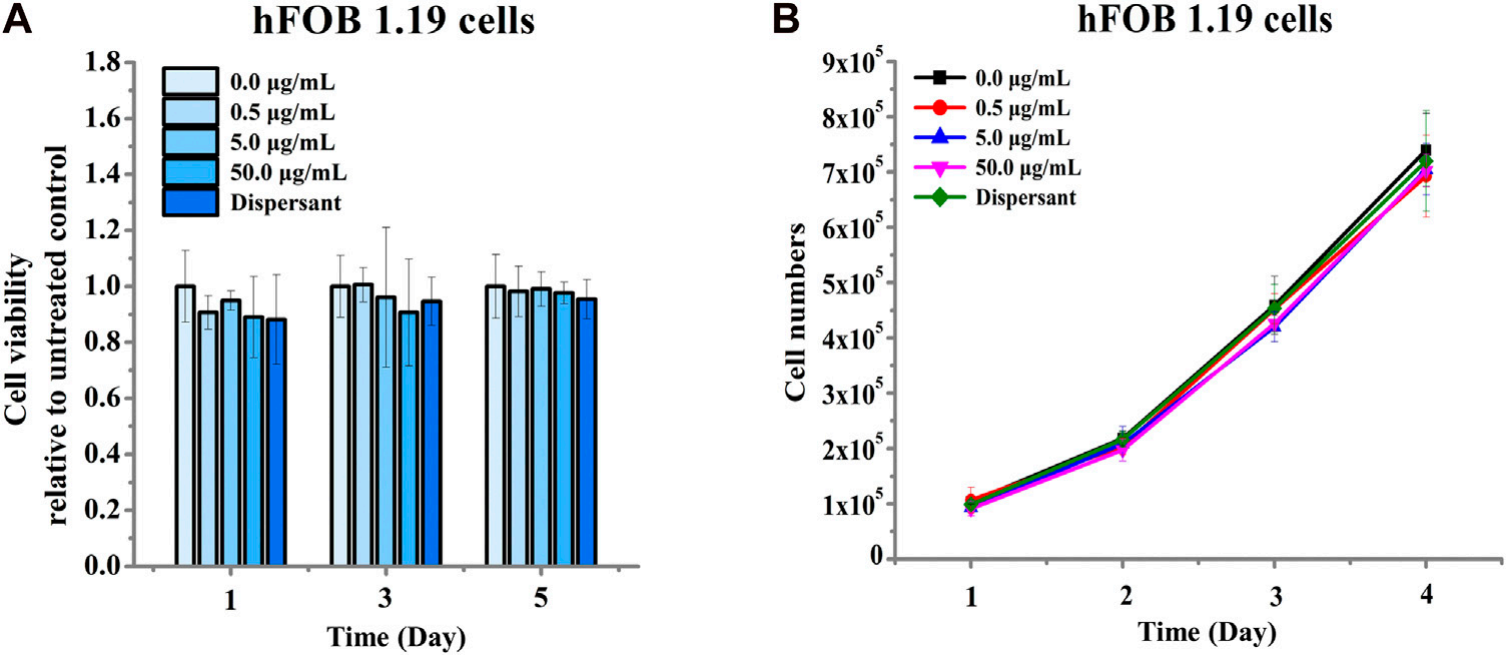
Cell viability of hFOB 1.19 cells treated with increasing concentrations of nano-HAP/β-TCP particles for the indicated days. **(A)** Viability of hFOB 1.19 cells assessed by the WST-1 assay after exposure to varying concentrations of nano-HAP/β-TCP particles. **(B)** Proliferation of hFOB 1.19 cells over time when treated with different concentrations of nano-HAP/β-TCP particles.

**FIGURE 3:**
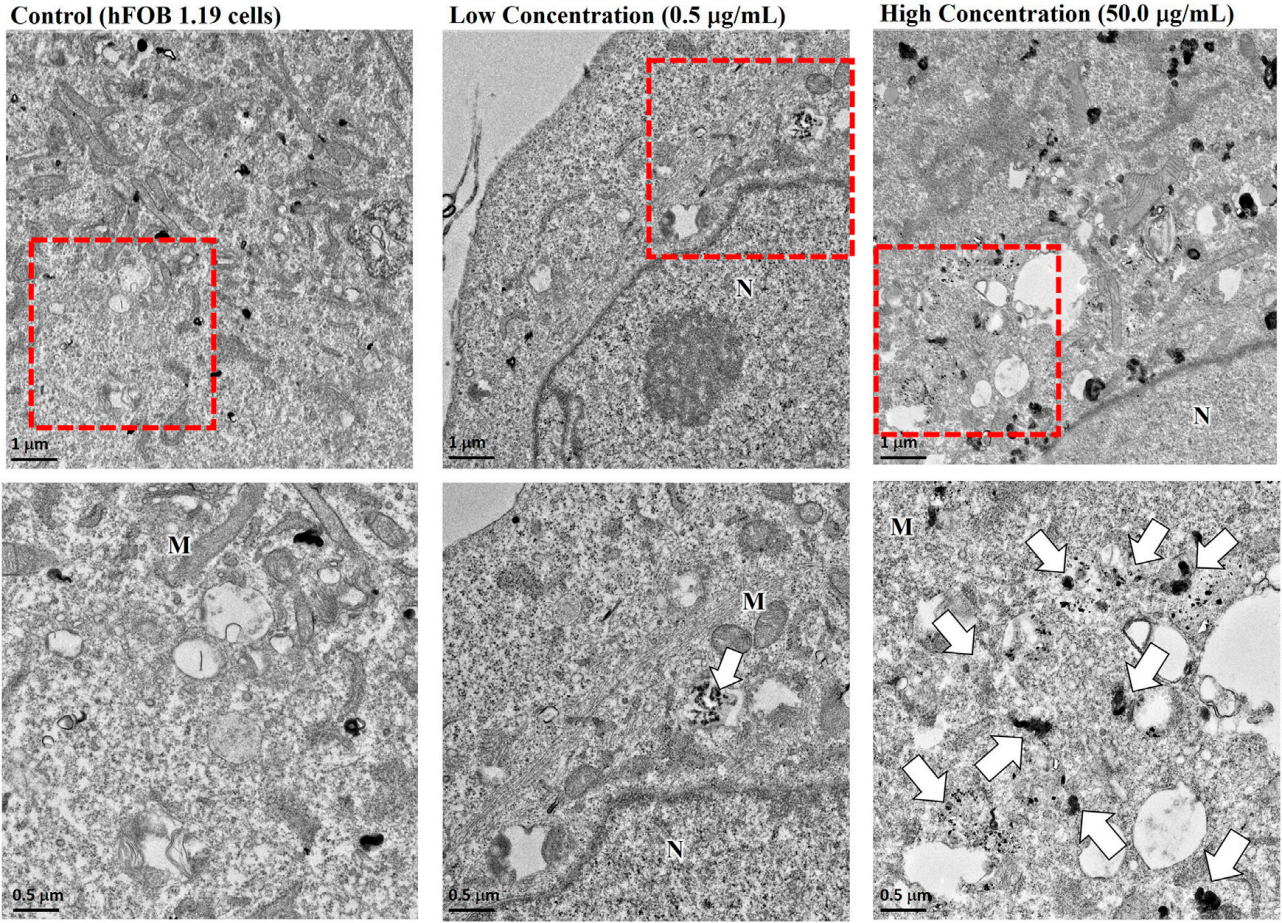
Transmission electron microscopy (TEM) images demonstrating the cellular uptake of nano-HAP/β-TCP particles by hFOB 1.19 cells when cells were treated with 0.5 or 50 μg/mL nano-HAP/β-TCP particles for 24 h. The red box areas in the upper panel are shown at higher magnification in the lower panel, and the arrows indicate the nano-HAP/β-TCP particles (M represents Mitochondria and N represents Nucleus). Scale bar: 1 μm (upper panel) or 0.5 μm (lower panel).

We next assessed the effects of nano-HAP/β-TCP on the viability and proliferation of the human osteoblastic hFOB 1.19 cells. Various concentrations of nano-HAP/β-TCP (0.5, 5, and 50 μg/mL) were tested, including treatment with the dispersant. The WST-1 assay performed on days 1, 3, and 5, and the cell count assay performed on days 1, 2, 3, and 4 post-treatment showed that both the dispersant and the nano-HAP/β-TCP treatments induced no significant impact on the viability and proliferation of osteoblasts (Figures 2A, B). These results suggest that nano-HAP/β-TCP exhibits negligible cytotoxicity toward hFOB 1.19 cells. However, we observed a significant increase in the intracellular ROS levels when treating cells with 50 μg/mL nano-HAP/β-TCP (Supplementary Figure S2A). Furthermore, we examined the cellular uptake of nano-HAP/β-TCP using TEM. As shown in Figure 3, nano-HAP/β-TCP was internalized into hFOB 1.19 cells and randomly dispersed in the cytoplasm at 24 h post-treatment when the cells were treated with either 0.5 or 50 μg/mL of the nanoparticles. These results demonstrate the ability of osteoblasts to uptake nano-HAP/β-TCP.

**FIGURE 4:**
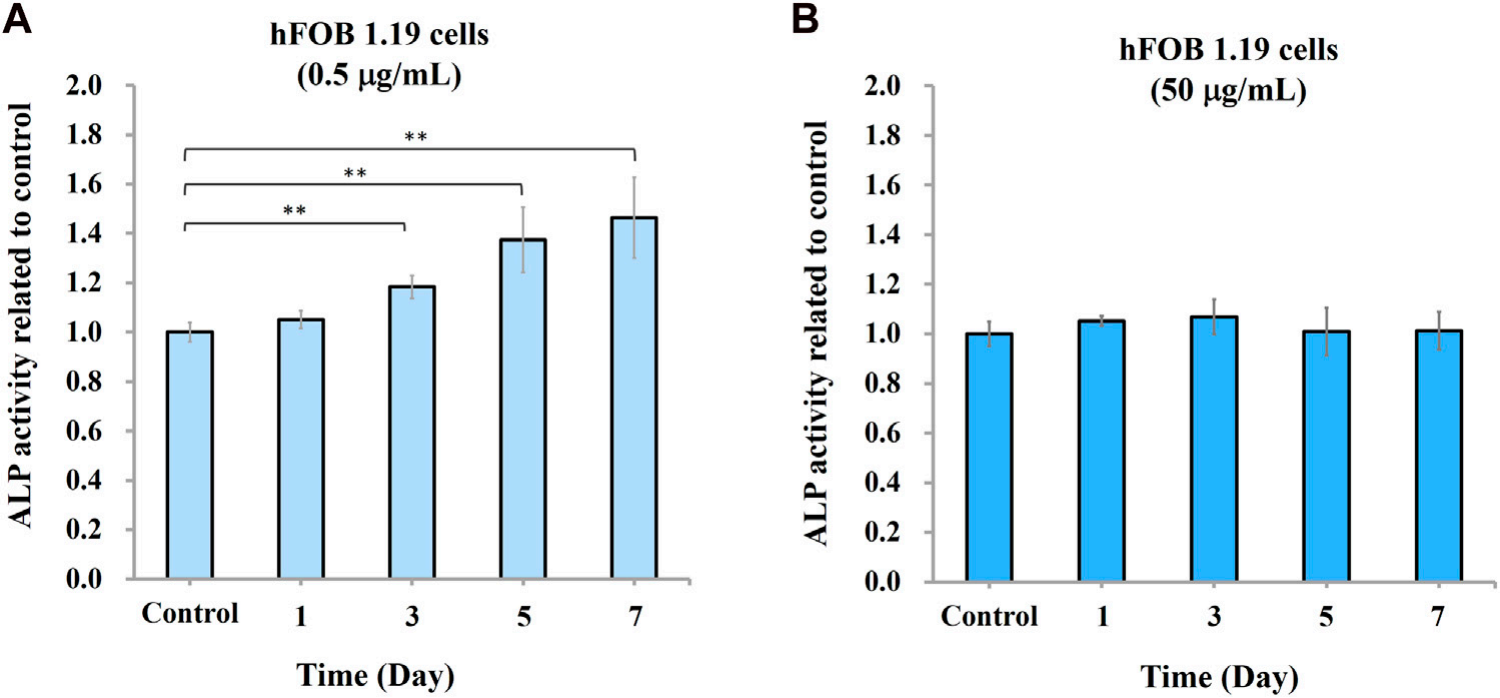
Osteogenic differentiation of hFOB 1.19 cells induced by nano-HAP/β-TCP particles. Measurement of alkaline phosphatase (ALP) activity in hFOB 1.19 cells treated with nano-HAP/β-TCP particles at the concentrations of **(A)** 0.5 μg/mL or **(B)** 50 μg/mL for the indicated days. * and ** indicate *p* < 0.05 and *p* < 0.01, respectively, compared to the control group.

**FIGURE 5:**
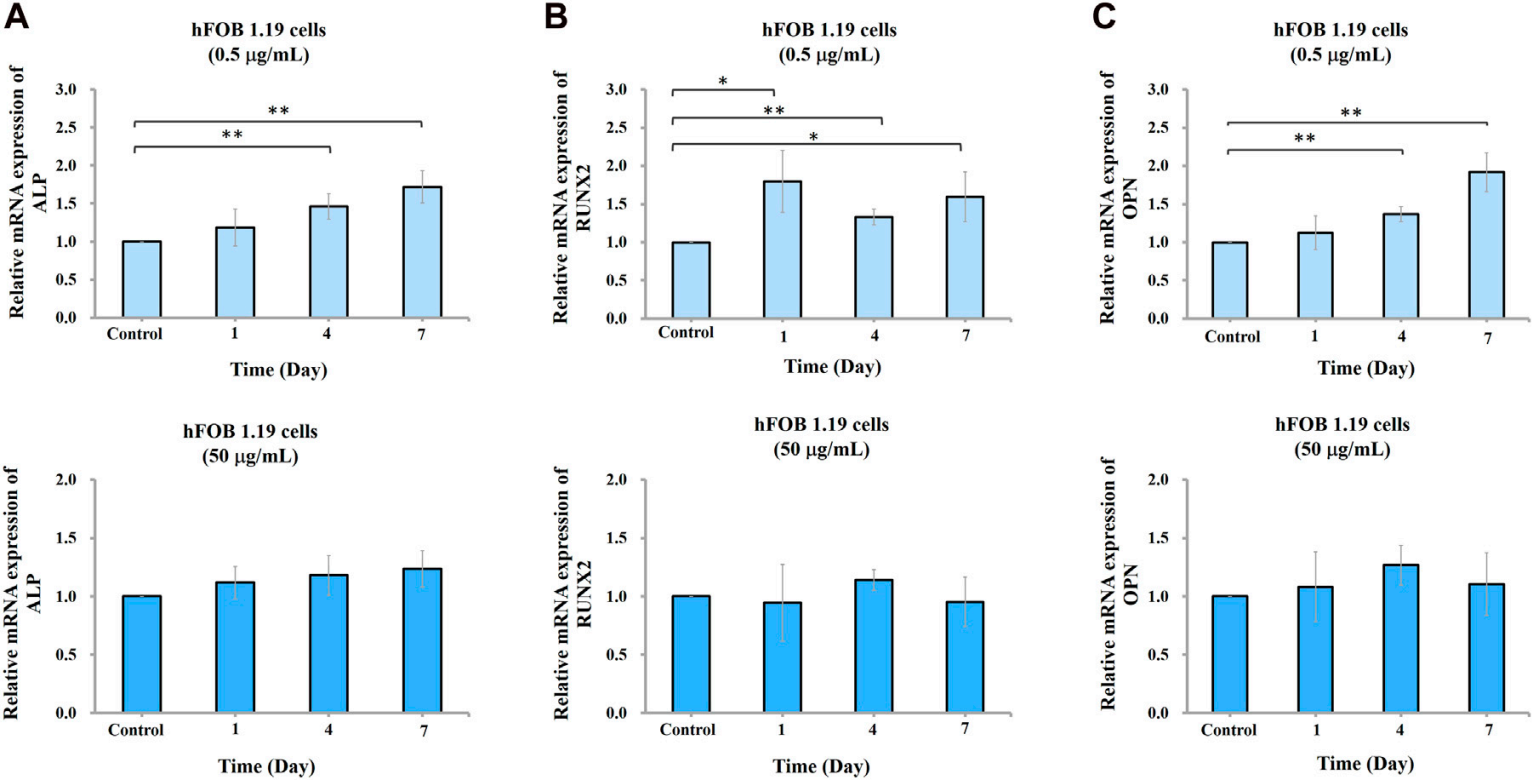
Gene expression of ALP, Runx2, and OPN in hFOB 1.19 cells treated with the indicated concentrations of nano-HAP/β-TCP particles. qRT-PCR analysis of **(A)** ALP, **(B)** Runx2, or **(C)** OPN gene expression levels performed on days 1, 4, and 7 post-treatment. * and ** indicate *p* < 0.05 and *p* < 0.01, respectively, compared to the control group.

To study the effect of nano-HAP/β-TCP on osteogenesis, we measured ALP activity and analyzed gene expression regulation in hFOB 1.19 osteoblasts in response to 0.5 and 50 μg/mL treatments (based on previous findings). Surprisingly, ALP activity was increased time-dependently when cells were treated with 0.5 μg/mL nano-HAP/β-TCP (Figure 4A). By contrast, this effect was not observed in cells treated with a significantly high and ROS-producible concentration of nano-HAP/β-TCP (50 μg/mL) (Figure 4B). Consistently, the mRNA expression levels of osteogenesis regulators, including ALP, Runx2, and OPN, were significantly upregulated in cells treated with 0.5 μg/mL nano-HAP/β-TCP compared with the controls. Meanwhile, a 50 μg/mL concentration of nano-HAP/β-TCP did not induce significant changes in the expression of those genes (Figures 5A–C). These findings indicate that lower concentrations of nano-HAP/β-TCP may be more effective in promoting osteogenesis than higher concentrations, which augment intracellular ROS production. Overall, our results imply that nano-HAP/β-TCP has the potential to enhance osteogenesis and provide favorable conditions for bone tissue regeneration.

### 3.3 Regulation of inflammatory cytokines produced by THP-1-derived macrophage by nano-HAP/β-TCP

**FIGURE 6:**
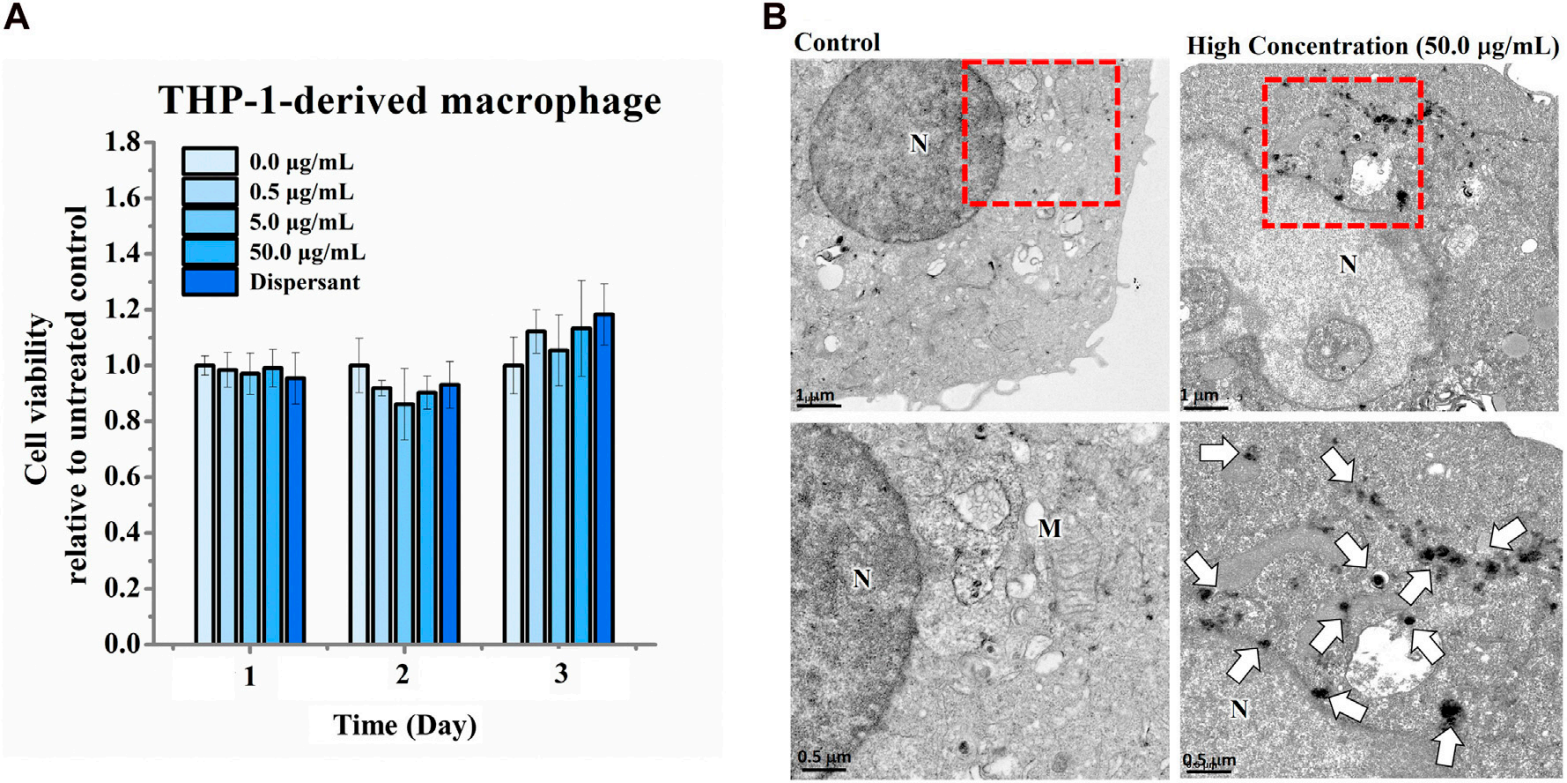
Cell viability of THP-1-derived macrophages treated with increasing concentrations of nano-HAP/β-TCP particles for the indicated days. **(A)** Cell viability measured with the WST-1 assay after THP-1-derived macrophages were exposed to various concentrations of nano-HAP/β-TCP particles. **(B)** TEM images illustrating the uptake of nano-HAP/β-TCP particles by THP-1-derived macrophages at a concentration of 50 μg/mL after 24 h of treatment. The red box areas in the upper panel are shown at higher magnification in the lower panel, and the arrows indicate the nano-HAP/β-TCP particles (M represents Mitochondria and N represents Nucleus). Scale bar: 1 μm (upper panel) or 0.5 μm (lower panel).

The ability of nano-HAP/β-TCP to promote osteogenesis raises a consideration regarding its role in inflammatory osteolysis. To address this, we examined the effects of nano-HAP/β-TCP on THP-1-derived macrophages, which can be activated by stimuli such as LPS to induce an inflammatory response. Activated macrophages produce pro-inflammatory cytokines, such as IL-1β, IL-6, and TNF-α, which promote osteoclast differentiation and bone resorption (Amarasekara et al., 2018). Our results showed that nano-HAP/β-TCP exposure did not significantly impact the viability of THP-1-derived macrophages, even at concentrations as high as 50 μg/mL (Figure 6A). Moreover, both the dispersant and the nano-HAP/β-TCP (0.5, 5, or 50 μg/mL) treatments did not affect the intracellular ROS levels in THP-1-derived macrophages (Supplementary Figure S2B). TEM images confirmed the successful uptake of nano-HAP/β-TCP into the cytoplasm when macrophages were treated with a 50 μg/mL concentration of these nanoparticles (Figure 6B).

**FIGURE 7:**
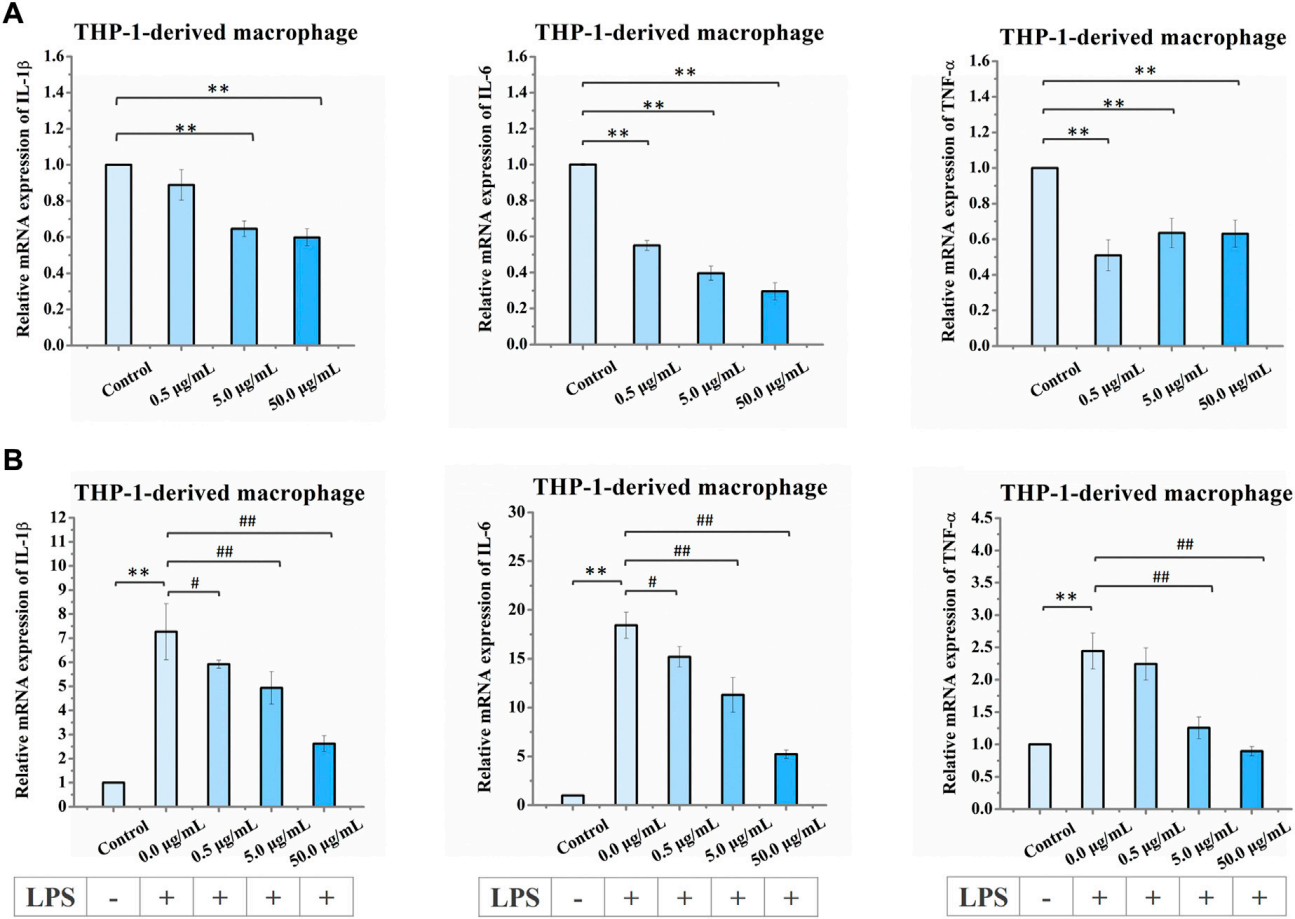
Relative mRNA expression levels in THP-1-derived macrophages after exposure to increasing concentrations of nano-HAP/β-TCP particles. **(A)** qRT-PCR analysis of IL-1β, IL-6, and TNF-α expression performed after 3 days of treatment with the indicated concentrations of nano-HAP/β-TCP particles (* and ** indicate *p* < 0.05 and *p* < 0.01, respectively, compared to the nano-HAP/β-TCP untreated group). **(B)** qRT-PCR analysis of IL-1β, IL-6, and TNF-α expression in THP-1-derived macrophages after LPS stimulation for 3 h followed by 1-day treatment with the indicated concentration of nano-HAP/β-TCP particles (** indicates *p* < 0.01, compared between the control and the LPS stimulation groups, while # and ## indicates *p* < 0.05 and *p* < 0.01, compared between various LPS stimulation groups, which were treated with different concentrations of nano-HAP/β-TCP particles).

Next, we examined the effects of nano-HAP/β-TCP treatment on the mRNA expression levels of the inflammatory genes IL-1β, IL-6, and TNF-α in THP-1-derived macrophages under both normal and LPS-induced inflammatory conditions. Under normal conditions, treatment of macrophages with nano-HAP/β-TCP at 5 or 50 μg/mL significantly diminished the gene expression levels of IL-1β, IL-6, and TNF-α (Figure 7A). Meanwhile, LPS stimulation augmented the expression of all these three inflammatory genes in THP-1-derived macrophages (Figure 7B). Furthermore, the LPS-induced elevation of IL-1β and IL-6 gene expression could be mitigated by treating cells with 0.5, 5, or 50 μg/mL nano-HAP/β-TCP, whereas that of TNF-α gene expression was alleviated by either a 5 or 50 μg/mL treatment with the nanoparticles. Importantly, the regulations of IL-1β, IL-6, and TNF-α mRNA expression levels by nano-HAP/β-TCP in LPS-stimulated macrophages were in a dose-dependent manner (Figure 7B).

**FIGURE 8:**
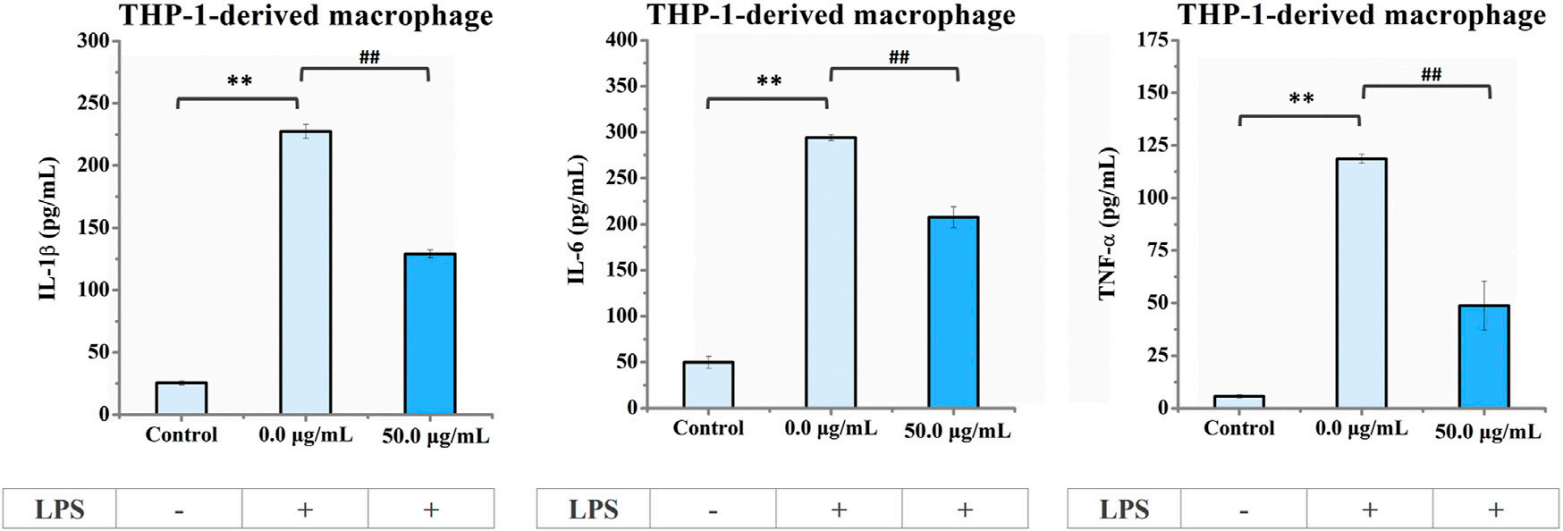
ELISA results of IL-1β, IL-6, and TNF-α cytokine secretion in the culture medium of THP-1-derived macrophages. Cells pre-stimulated with LPS for 3 h followed by treatment with nano-HAP/β-TCP particles for 24 h secreted lower amounts of pro-inflammatory factors (IL-1β, IL-6, or TNF-α) than those stimulated with LPS alone. ** indicates *p* < 0.01, compared between the control and LPS stimulation groups, and ## indicates *p* < 0.01, compared between the LPS stimulation groups with or without nano-HAP/β-TCP treatment.

In addition, we analyzed the extracellular levels of the IL-1β, IL-6, and TNF-α cytokines in LPS-stimulated macrophages with or without 50 μg/mL nano-HAP/β-TCP treatment. In line with the gene expression results shown in Figure 7B, the extracellular cytokine levels of IL-1β, IL-6, and TNF-α were dramatically elevated when macrophages were stimulated with LPS but could be diminished in the presence of 50 μg/mL nano-HAP/β-TCP (Figure 8). Collectively, these results indicate that nano-HAP/β-TCP may have an impact on macrophage inflammatory activity under both normal and LPS-exposed conditions.

### 3.4 Recovery of impaired osteogenesis ability triggered by LPS-stimulated macrophage-conditioned medium with nano-HAP/β-TCP particles

**FIGURE 9:**
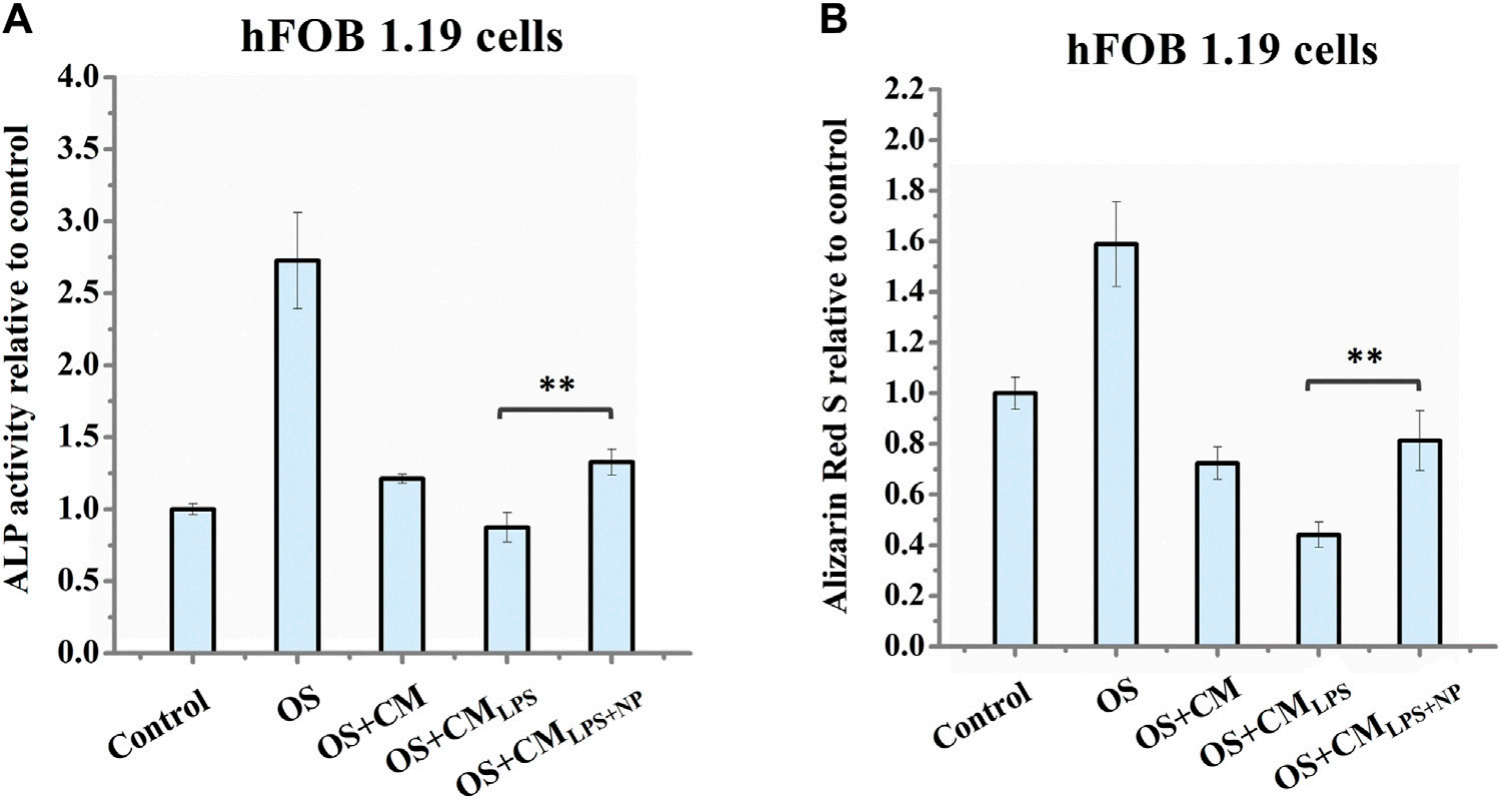
**(A)** Measurement of ALP activity in hFOB 1.19 cells after treatment for 7 days and **(B)** Alizarin Red S staining of hFOB 1.19 cells after treatment for 14 days. OS: osteogenic differentiation medium supplemented with dexamethasone, β-glycerol phosphate, and ascorbate-2-phosphate; CM: conditioned medium collected from untreated THP-1-derived macrophages; CM_LPS_: conditioned medium collected from THP-1-derived macrophages treated with LPS for 27 h; CM_LPS+NP_: conditioned medium collected from macrophages pre-stimulated with LPS for 3 h, followed by being treated with 50 μg/mL nano-HAP/β-TCP particles for 24 h ** indicates *p* < 0.01, compared between the OS + CM_LPS_ and the OS + CM_LPS+NP_ groups.

To study the potential of nano-HAP/β-TCP-mitigated inflammatory response for modulating bone regeneration, we examined the effect of conditioned medium from LPS-stimulated macrophages with or without nano-HAP/β-TCP treatment on the osteogenic ability of hFOB 1.19 osteoblasts. We collected conditioned medium from untreated THP-1-derived macrophages (CM) or macrophages pretreated with LPS for 3 h, followed by being treated with nano-HAP/β-TCP (CM_LPS+NP_) for 24 h or no additional treatment (CM_LPS_). The resulting conditioned medium (CM, CM_LPS_, or CM_LPS+NP_) was then applied to hFOB 1.19 osteoblasts, which were cultured in an osteogenic differentiation medium (OS) supplemented with dexamethasone, ascorbate-2-phosphate, and β-glycerol phosphate. hFOB 1.19 cells were cultured in macrophage-conditioned media for 7 days for the ALP activity assay or 14 days for the mineralization assay. The hFOB 1.19 cells cultured parallelly in OS medium only were used as the positive control (OS), while cells cultured in normal growth medium were used as the negative control (control). As shown in Figures 9A, B, the ALP activity and the amount of Alizarin Red S staining in the OS group was significantly increased as compared to the control group, indicating a successful induction of osteogenesis when hFOB 1.19 cells were maintained in the OS medium. Moreover, both the ALP activity (Figure 9A) and the amount of Alizarin Red S staining (Figure 9B) in the OS + CM_LPS+NP_ group were significantly higher than those in the OS + CM_LPS_ group and were comparable with those in the OS + CM group. These data imply that the conditioned medium derived from LPS-stimulated macrophages attenuates the ALP activity and mineralization of human osteoblasts, whereas these effects could be counteracted in the presence of BCP nanomaterials.

## 4 Discussion

In recent years, micron-sized BCP materials have been proven to be safe and effective for clinical applications, particularly as scaffolds for tissue repair. These materials provide essential support for tissue growth and aid in the regeneration of damaged tissues. In this study, we employed a wet polish method using a nanobead mill to transform a commercial micron-scaled BCP material, known as Bicera (FDA regulation number: K172237), into nanoscaled BCP material. Bicera is an FDA-approved product known for its safety, effectiveness, biocompatibility, and osteoinductive properties (Chen et al., 2017).

The field of biomaterial development has evolved from a focus solely on biocompatibility to an emphasis on robust immunomodulatory characteristics. Yang et al. highlighted the osteogenic potential of hydroxyapatite nanoparticles in human mesenchymal stem cells (hMSCs) *in vitro*, with smaller-sized nanoparticles (50 and 100 nm) exhibiting a higher differentiation rate, potentially due to different concentrations of released Ca^2+^ (Yang et al., 2018). Another study by Fatima et al. demonstrated that selenium nanoparticles may increase the protein expression of p-JNK and FOXO3 via the induction of antioxidant enzymes, thereby promoting the differentiation of mesenchymal stem cells to osteoblasts (Fatima et al., 2021). Stem cells with polycaprolactone/gelatin/hydroxyapatite nanocomposite scaffolds enhance bone repair *in vitro* and *in vivo* (Sattary et al., 2022). Therefore, in addition to ensuring safety, ideal nanoparticles should create a suitable microenvironment for bone tissue repair and differentiation of bone marrow mesenchymal stromal cells (Franz et al., 2011). In this study, we evaluated the biocompatibility, bone regeneration potential, and immunomodulatory abilities of the nanoparticulate BCP materials HAP/β-TCP with a size of 100 nm prepared by the nanogrinding dispersion technique.

There are four primary methods for synthesizing calcium phosphate materials: dry chemical precipitation, wet chemical synthesis, high-temperature processing, and synthesis from biogenic resources (Basu and Basu, 2019). The precipitation method used to prepare calcium phosphate nanoparticles often results in needle-like structures with varying aspect ratios. The aspect ratio of nanoparticles, ranging from 1 for spherical particles to near zero for nanotubes, influences their toxicity. Higher aspect ratios are associated with increased toxicity. Needle-like nanoparticles exhibit poor biocompatibility, cytotoxicity, and even pulmonary toxicities (Lippmann, 1990; Muller et al., 2005; Li et al., 2015; Egbuna et al., 2021). Although high-temperature and high-pressure methods can modify needle-like structures, they introduce complexity and uncertainty. In addition, the precipitation-based method frequently results in nanoparticle aggregation, which can impact bioreactivity (Motskin et al., 2009; Motskin et al., 2011; Muller et al., 2014; Wang et al., 2019).

In this study, we addressed these challenges using a polymeric carboxylic acid dispersant to prevent nanoparticle aggregation. In addition, the ratio of BCP influences its biological properties, such as cellular degradation and protein adsorption (Yamada et al., 1997). An HAP/β-TCP ratio of 60/40 is a suitable option for enhancing the degradation rate of HAP (Jensen et al., 2009). This specific ratio, combined with the use of 100 μm zirconia beads and the dispersant, allowed us to grind micron-sized BCP materials into particles with an average size of approximately 100 nm. This process is suitable for industrial-scale production and produces granular-shaped nanoparticles, as confirmed by electron microscopy. The use of a dispersant imparts a negative charge to the nanoparticle surfaces, preventing particle aggregation and maintaining their nanoscale properties over extended periods.

Previous studies have indicated that nanoparticles may impact cell activity and viability by modulating the reactive oxygen species (ROS) response to influence the osteogenic process (Callaway and Jiang, 2015; Sheppard et al., 2022). While some studies suggest that nanoparticles may stimulate osteoblast differentiation through antioxidative processes (Fatima et al., 2021), our study reveals that treatment of hFOB 1.19 cells with a high concentration of nano-HAP/β-TCP particles (50 μg/mL) increases intracellular ROS levels but induce no significant effects on osteogenesis and the expression of osteogenesis regulators (Figure 4; Figure 5). By contrast, a low concentration of nanoparticles (0.5 μg/mL) enhances osteogenic activity without triggering an associated increase in ROS levels. Our data indicate that the osteogenic process triggered by lower concentrations of nano-HAP/β-TCP particles may not be related to ROS-mediated signaling pathways, warranting further investigation into the underlying mechanisms.

The bone recovery process typically consists of different phases, including the inflammatory, proliferative, and remodeling phases (Wen et al., 2023). In the inflammatory phases, macrophages play an important role by differentiating into subtypes that induce pro- or anti-inflammation (Nich et al., 2013; Wen et al., 2023). Ideal osteogenic materials that can adapt to the local microenvironment and stimulate osteogenesis still need to be developed. Mesoporous silica nanoparticles (MSNs) have shown a positive immunomodulatory effect on macrophages, leading to the inhibition of inflammation (Zhang et al., 2018). Moreover, studies involving rapamycin-loaded silica nanocarriers have demonstrated their capacity to trigger autophagy-mediated M2 macrophage polarization, enhancing bone regeneration through the osteogenic differentiation of bone marrow mesenchymal stromal cells (Zhang et al., 2023). Other possible mechanisms related to the activation of autophagy have been proposed. Wang et al. explored the influence of nano-HAP on osteoblast differentiation and found that dispersed nano-HAP induced autophagy in a dose-dependent manner through the potential involvement of the mTOR signaling pathway (Wang et al., 2019). Overall, this evidence indicates that modulating the immune environment can significantly enhance osteogenesis, highlighting the potential of nano-immunotherapy effects of nanomaterials. In our study, we observe that nano-HAP/β-TCP particles can modulate inflammation in THP-1-derived macrophages, either with or without LPS stimulation (Figure 7; Figure 8). Furthermore, nano-HAP/β-TCP particles could reverse the effects of LPS-stimulated macrophage-conditioned medium on reducing the osteogenic potential of hFOB 1.19 cells (Figure 9). Collectively, our results suggest the immunomodulatory capacities of nano-HAP/β-TCP particles, which can modulate the microenvironment by mitigating macrophage-produced inflammatory mediators, thereby improving the osteogenic ability of human fetal osteoblastic cells. These findings underscore the significant potential for the clinical use of nanoparticles as effective biomaterials (Ghahremani et al., 2018; Kefayat et al., 2019a; Kefayat et al., 2019b) and in the prevention of inflammatory osteolysis.

## 5 Conclusion

In this study, we introduced a novel approach for synthesizing nanoscaled hydroxyapatite (HAP) and β-tricalcium phosphate (β-TCP) particles using a polymeric carboxylic acid as the dispersant. Our method successfully produced granular-shaped BCP nanoparticles with particle sizes averaging approximately 100 nm. These nano-HAP/β-TCP particles exhibit excellent biocompatibility and demonstrate the unique capability to modulate the local immune system while promoting osteogenesis, as evidenced by our *in vitro* experiments. Although our findings are promising, the translation of these nanoparticles into clinical practice will necessitate further investigation. Future studies will focus on *in vivo* experiments to compare the performance and efficacy of these nanoscaled BCP materials with those of conventional osteogenic materials. These endeavors will pave the way for the potential use of these nanoparticles in bone regeneration and open new avenues for advanced biomaterials with immunomodulatory properties.

## Data Availability

The original contributions presented in the study are included in the article/Supplementary Material, further inquiries can be directed to the corresponding authors.
