# Supplementary material for: Nanoscaled biphasic calcium phosphate modulates osteogenesis and attenuates LPS-induced inflammation
Source: Front Bioeng Biotechnol. 2023 Nov 29;11:1236429. doi: 10.3389/fbioe.2023.1236429 (PMC10716545; doi:10.3389/fbioe.2023.1236429)
Supplement: Supplementary file 1 [file DataSheet2.PDF]

Size Report - 2 Page Example

Sample Details

**Sample Name:**	bone powder		
**Project Name:**	biolab		
**Date and Time:**	Tuesday, July 28 2020 12:03:01 PM		
**Type:**	Size	**Result Source:**	Edited
**Cell Name:**	DTS0012	**Temperature (°C):**	25
**Material Name:**	Polystyrene latex	**Dispersant Name:**	Water
**Material RI:**	1.59	**Dispersant RI:**	1.33
**Material Absorption:**	0.01	**Dispersant Viscosity (cP):**	0.887
		**Dispersant Dielectric Constant:**	78.5

Size Distribution by Intensity

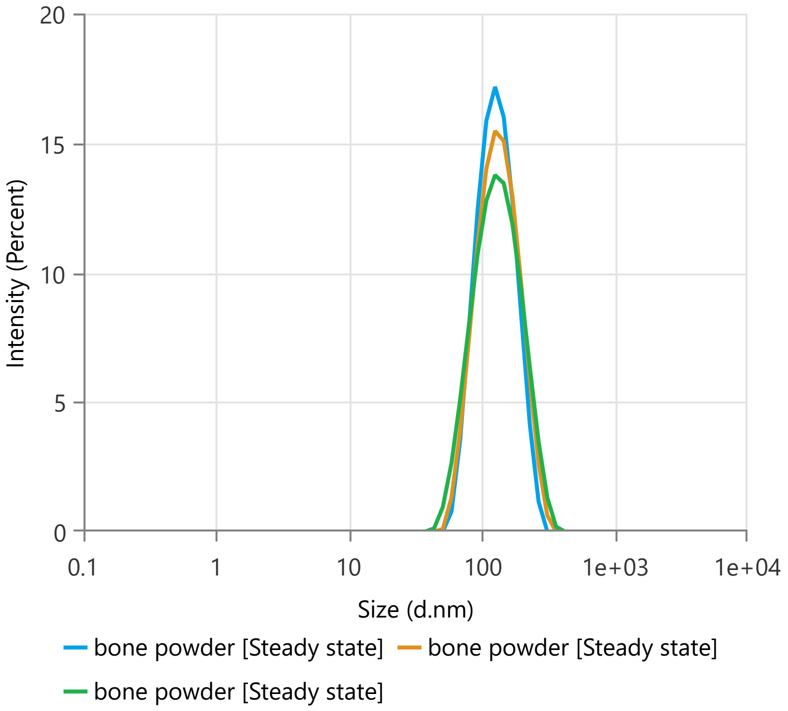

Size Distribution by Volume

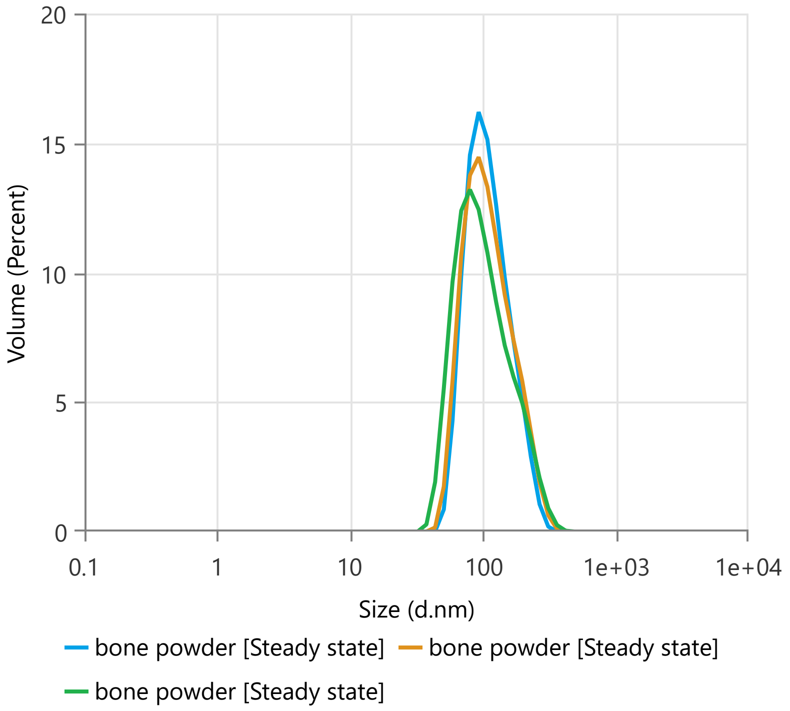

Size Report - 2 Page Example

Correlogram

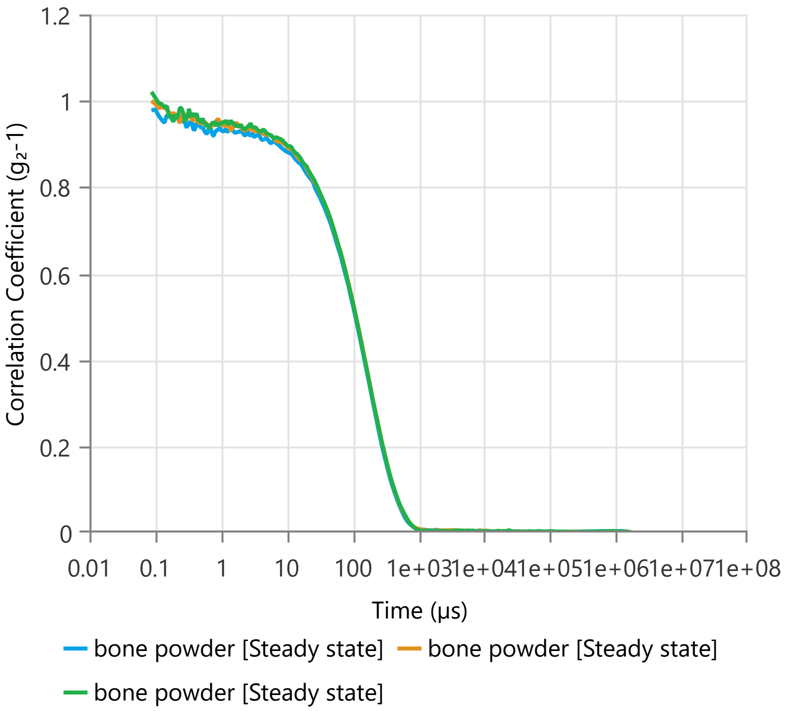

Cumulants Fit

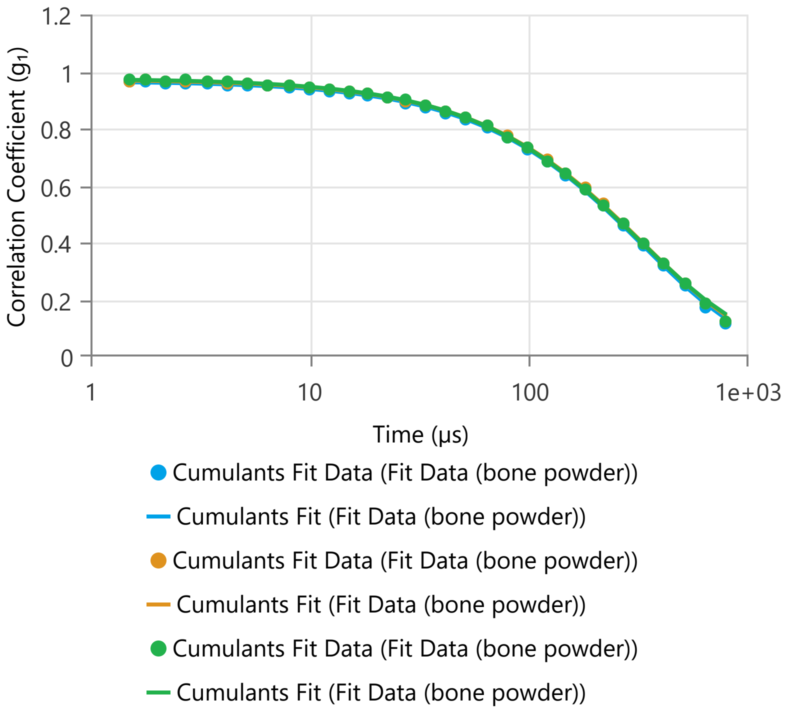

Data Quality Guidance

bone powder
No data quality issues detected.

bone powder
No data quality issues detected.

bone powder
No data quality issues detected.

Statistics Table

Name	Mean	Standard Deviation	RSD	Minimum	Maximum
Z-Average (nm)	116.7	1.438	1.232	115.1	117.8
Polydispersity Index (PI)	0.1446	0.02483	17.17	0.1191	0.1686
Intercept	0.9713	0.004392	0.4522	0.9665	0.975
Fit Error	0.001569	0.0001823	11.62	0.001393	0.001757
In Range (%)	96.97	0.2212	0.2281	96.71	97.12
Peak One Mean by Intensity (nm)	137.6	3.615	2.626	133.5	139.8

Size Report - 2 Page Example

Parameter List

**Instrument Serial Number**	:	MAL1233483
**Software Version**	:	1.3.1.7
**Method Path**	:	
