# Supplementary figures and images for: Nanoscaled biphasic calcium phosphate modulates osteogenesis and attenuates LPS-induced inflammation
Source: Front Bioeng Biotechnol. 2023 Nov 29;11:1236429. doi: 10.3389/fbioe.2023.1236429 (PMC10716545; doi:10.3389/fbioe.2023.1236429)

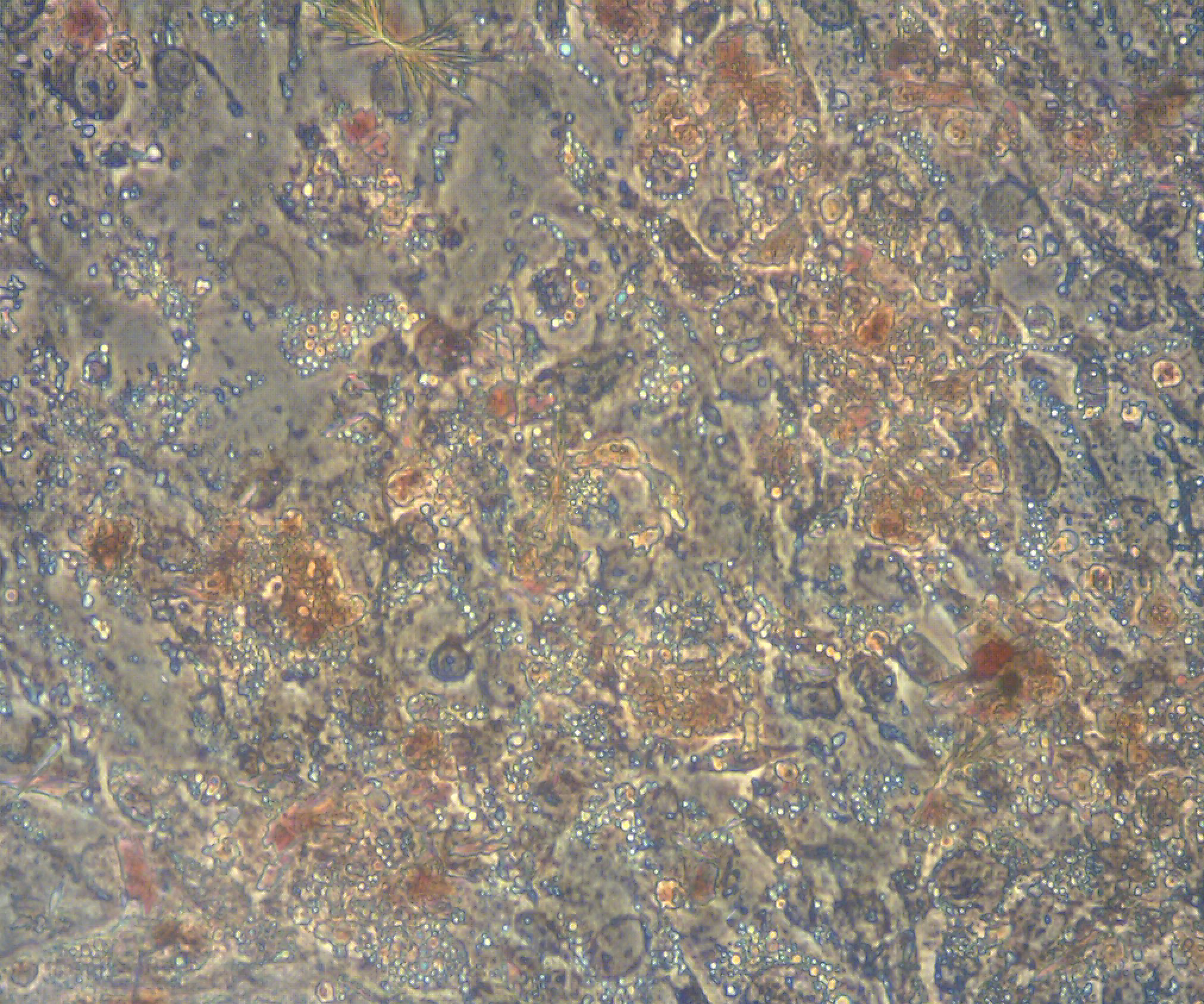

Supplement: Supplementary file 2 [file DataSheet3.ZIP › Alizarin Red S Staining_final/Control.jpg]

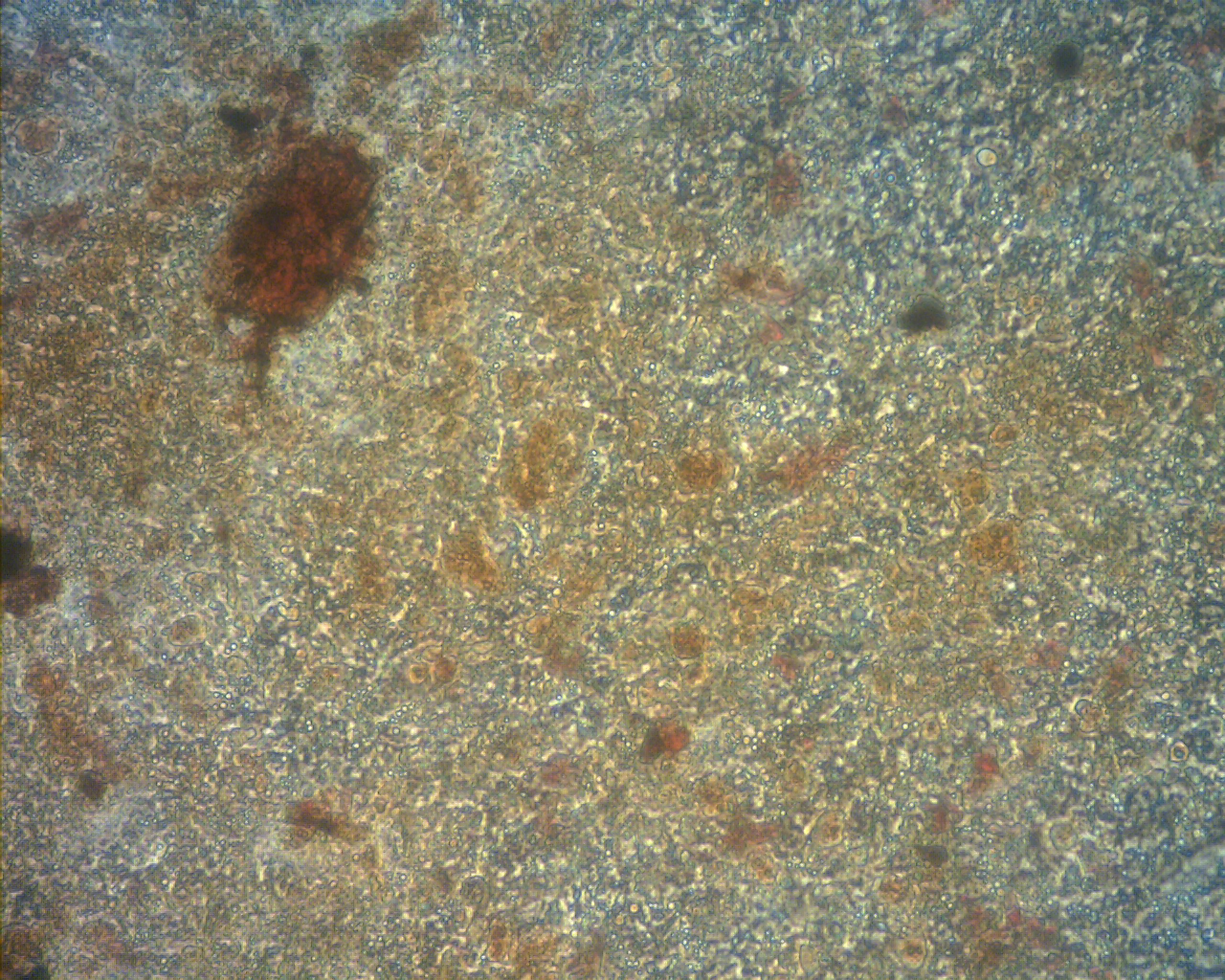

Supplement: Supplementary file 2 [file DataSheet3.ZIP › Alizarin Red S Staining_final/OS.jpg]

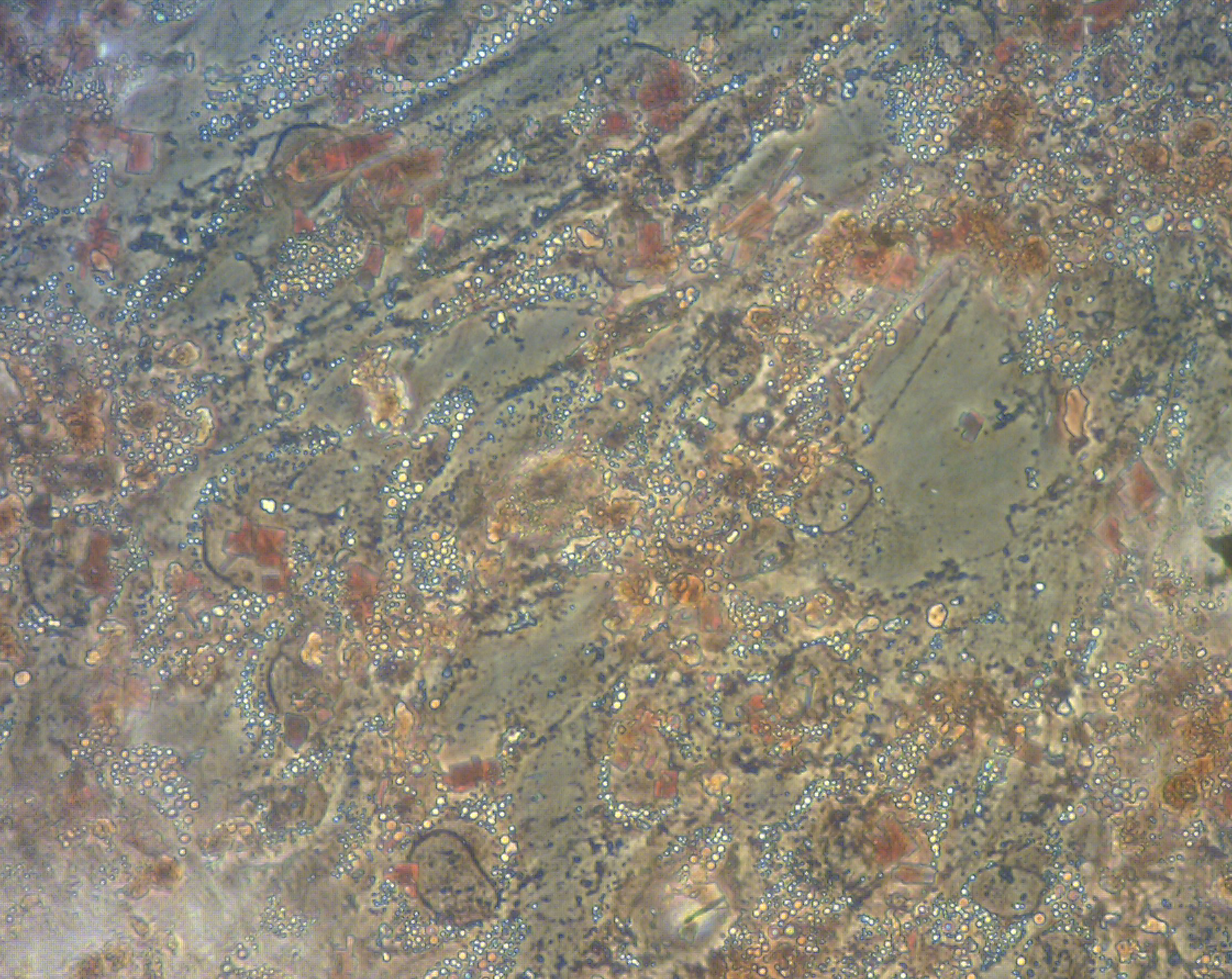

Supplement: Supplementary file 2 [file DataSheet3.ZIP › Alizarin Red S Staining_final/OS_CM.jpg]

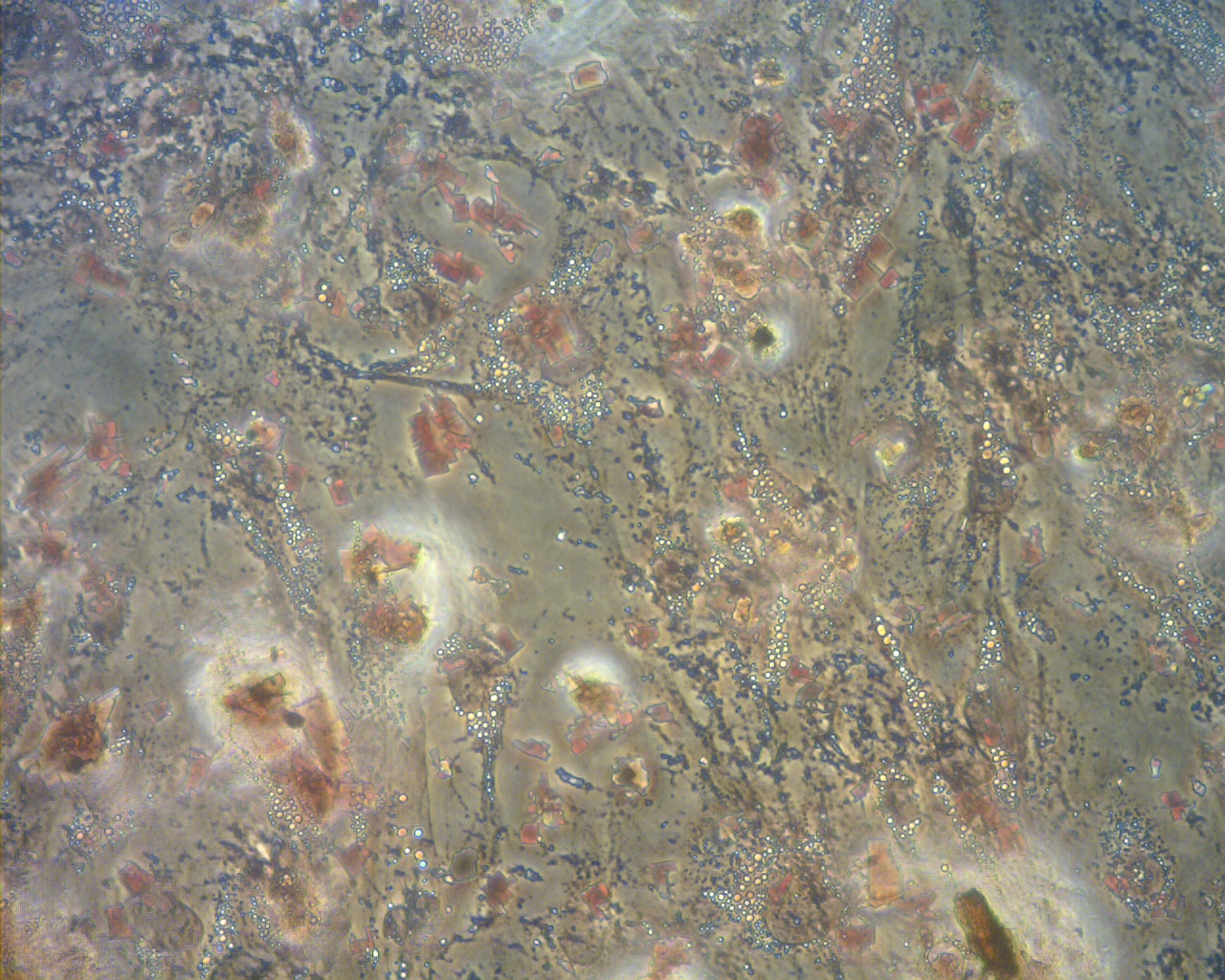

Supplement: Supplementary file 2 [file DataSheet3.ZIP › Alizarin Red S Staining_final/OS_LPS_CM.jpg]

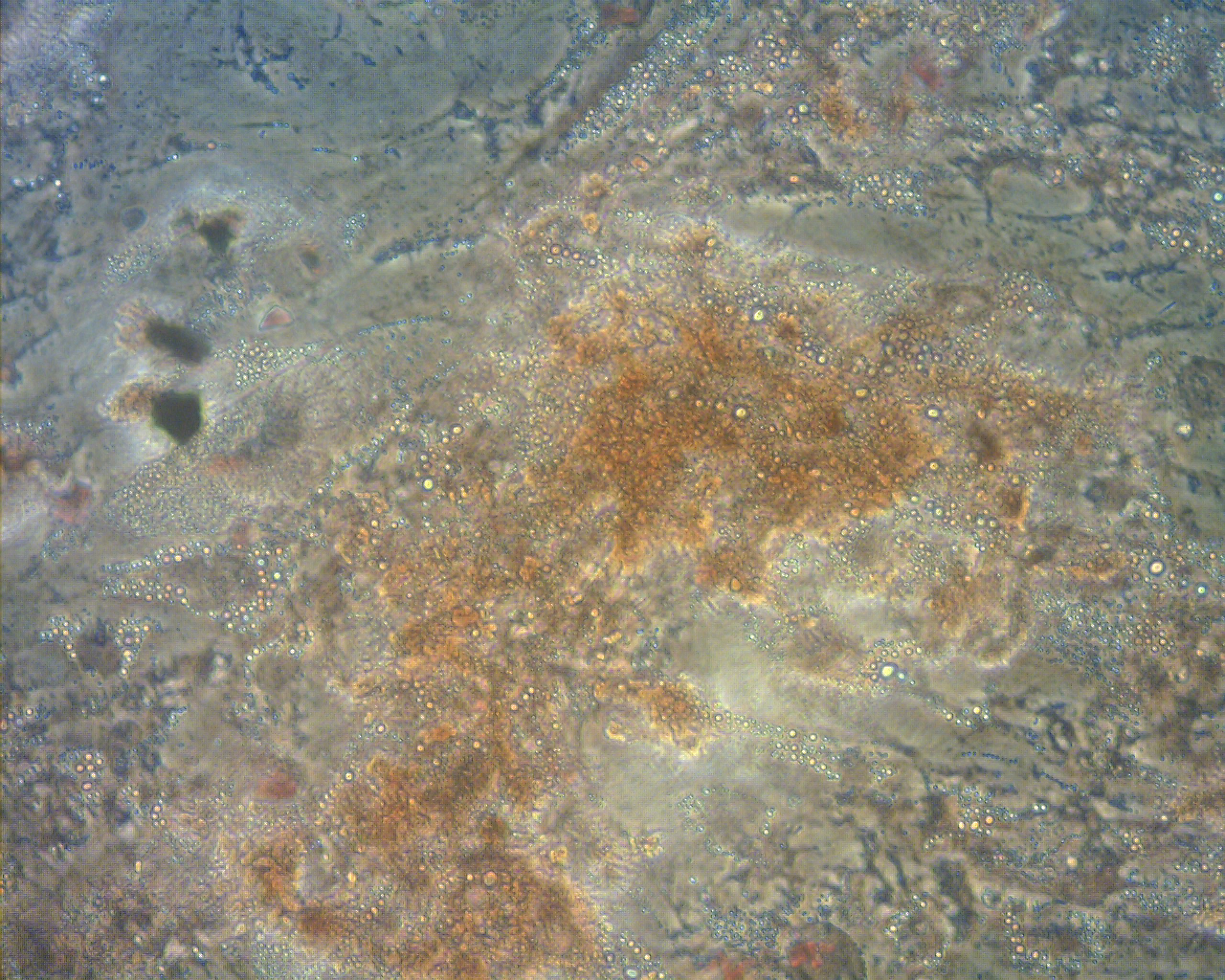

Supplement: Supplementary file 2 [file DataSheet3.ZIP › Alizarin Red S Staining_final/OS_LPS_NP_CM.jpg]

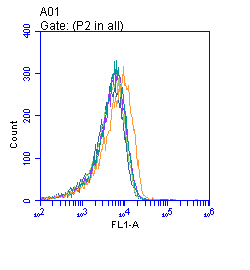

Supplement: Supplementary file 3 [file DataSheet4.ZIP › ROS_final/hFOB_ROS.png]

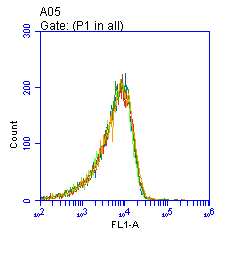

Supplement: Supplementary file 3 [file DataSheet4.ZIP › ROS_final/THP1_ROS.png]

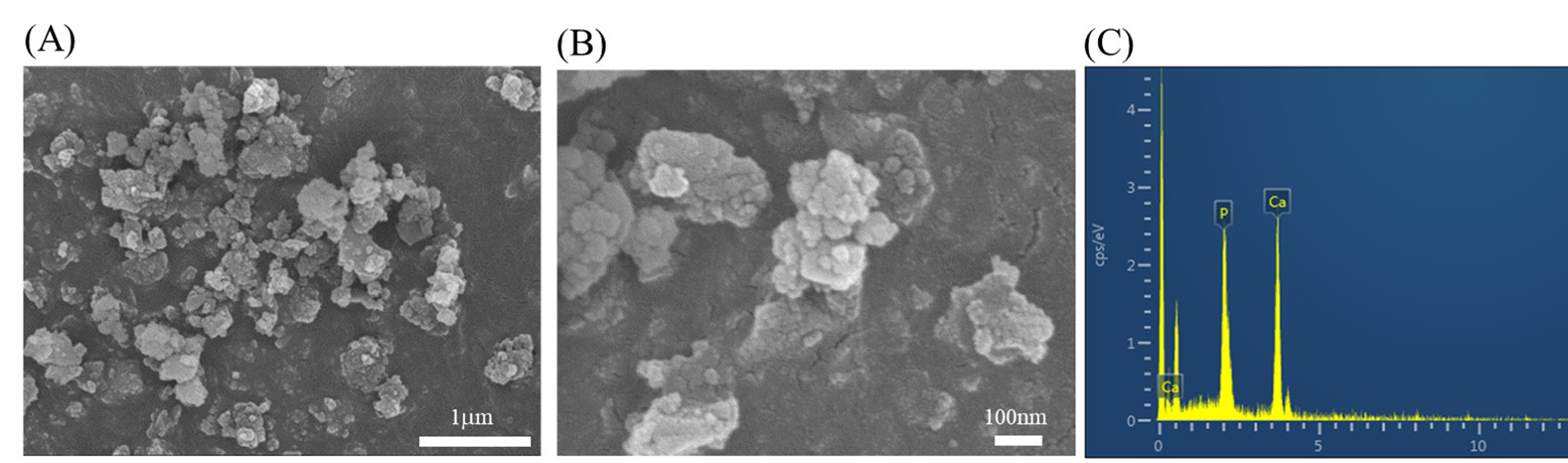

Supplement: Supplementary file 4 [file Image1.JPEG]

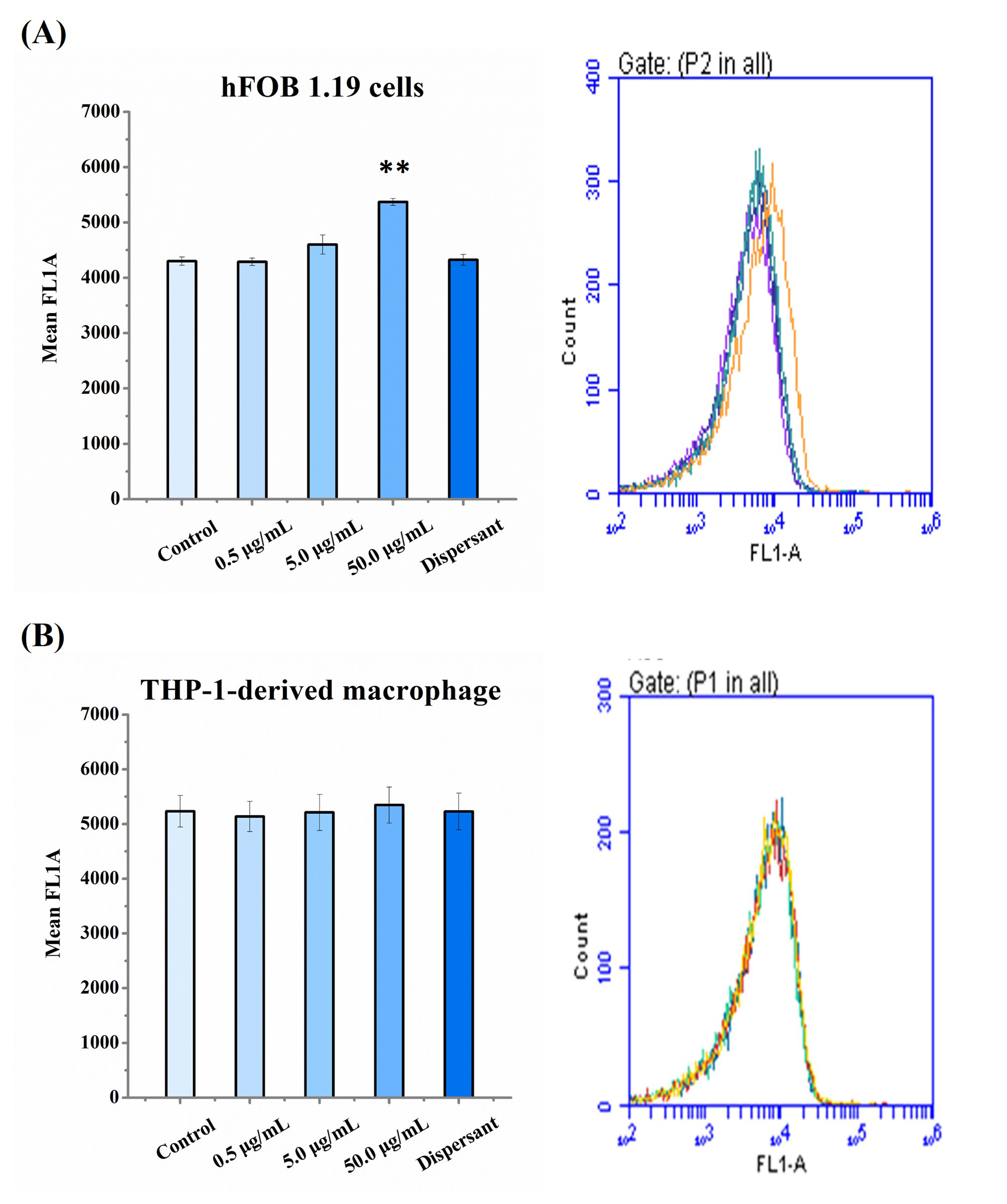

Supplement: Supplementary file 5 [file Image2.JPEG]

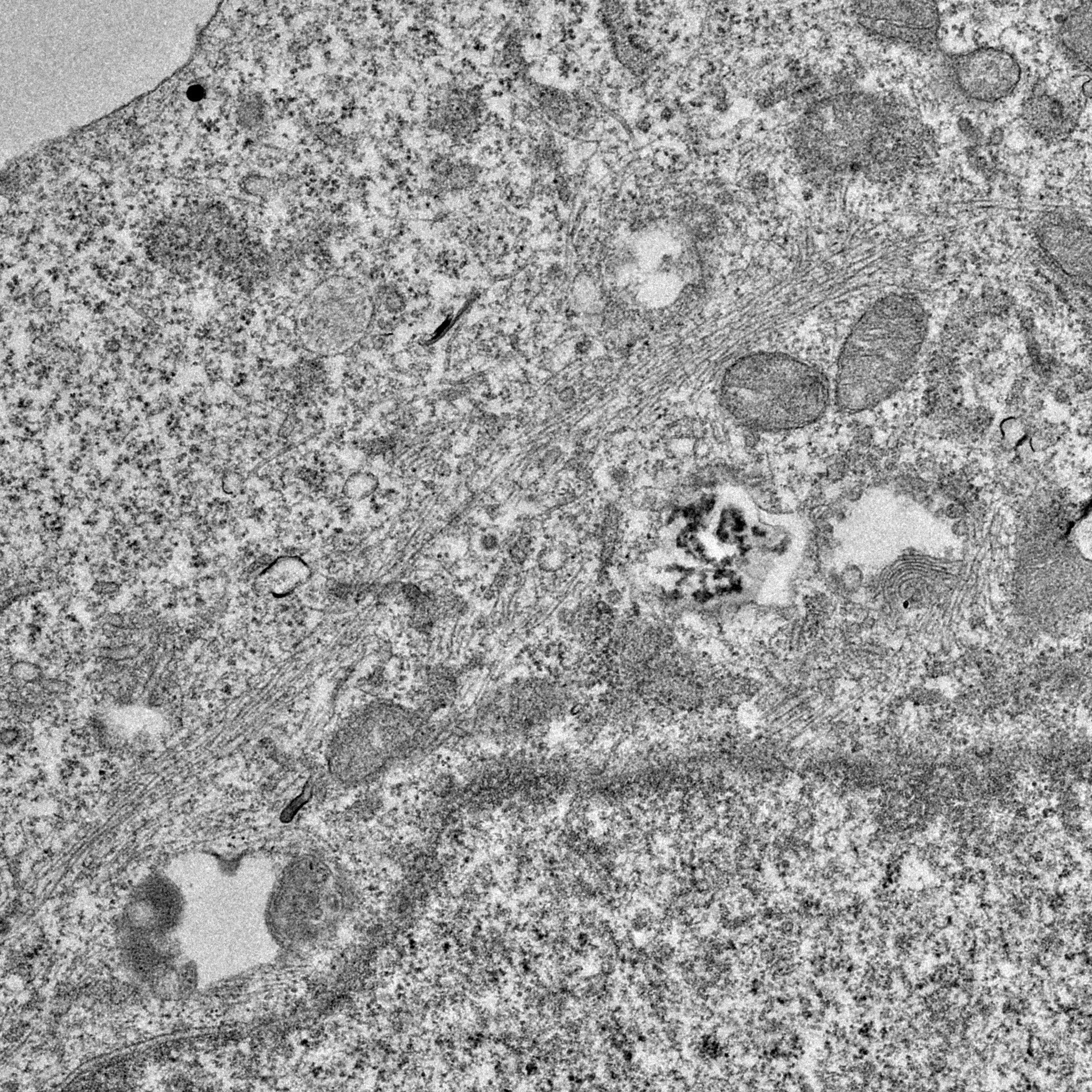

Supplement: Supplementary file 6 [file DataSheet6.ZIP › TEM_final/hFOB_0.5ug_highmagnification.jpg]

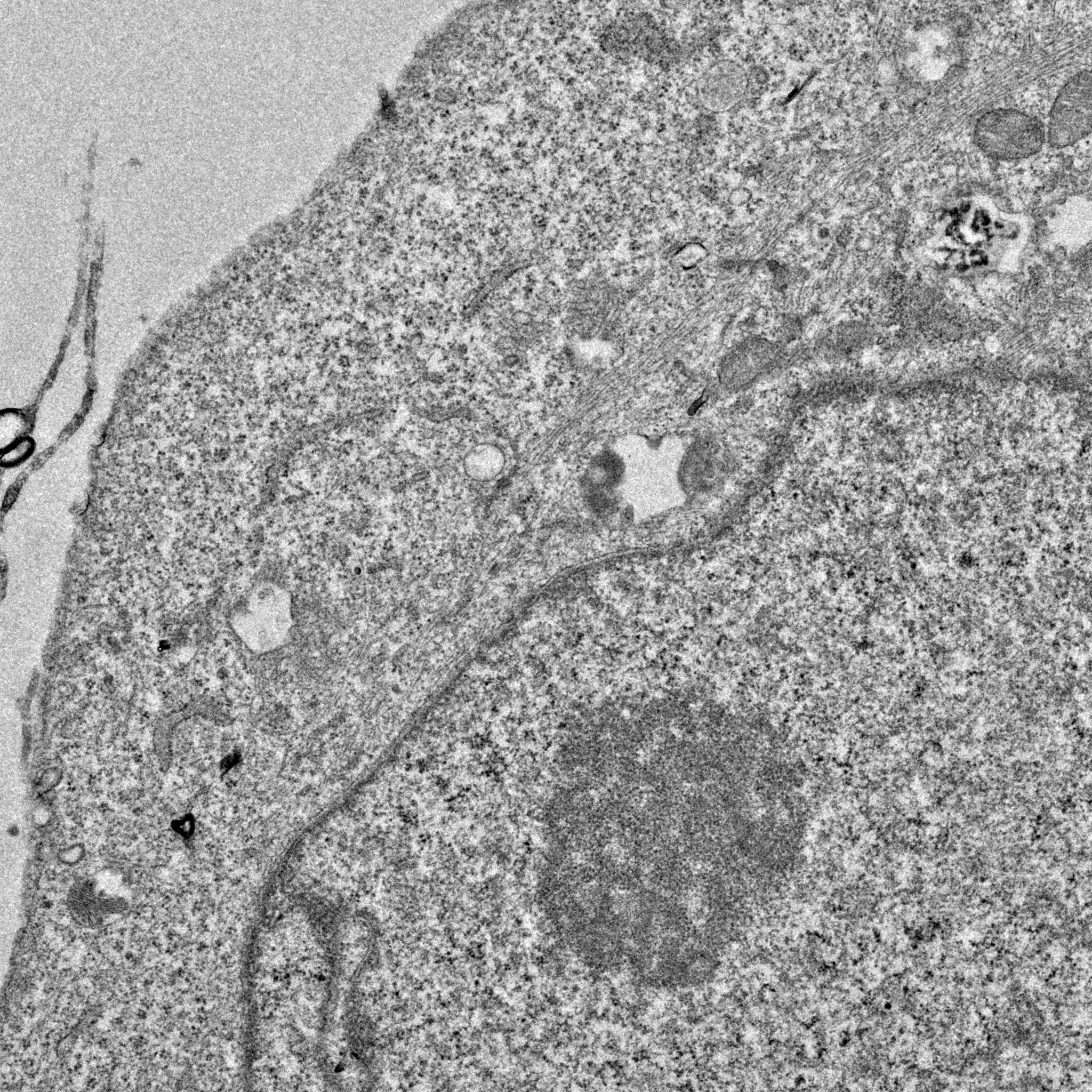

Supplement: Supplementary file 6 [file DataSheet6.ZIP › TEM_final/hFOB_0.5ug_low magnification.jpg]

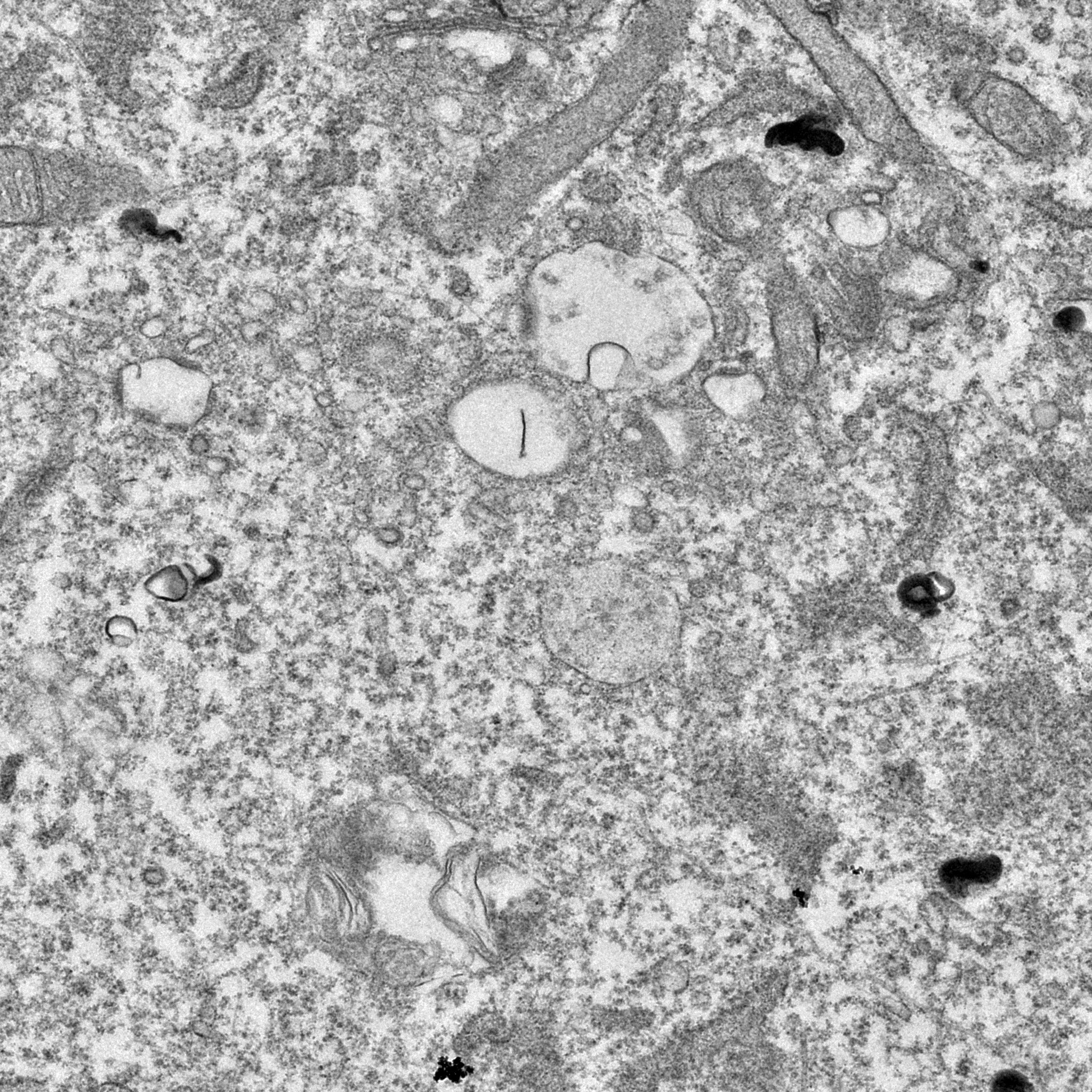

Supplement: Supplementary file 6 [file DataSheet6.ZIP › TEM_final/hFOB_0ug_highmagnification.jpg]

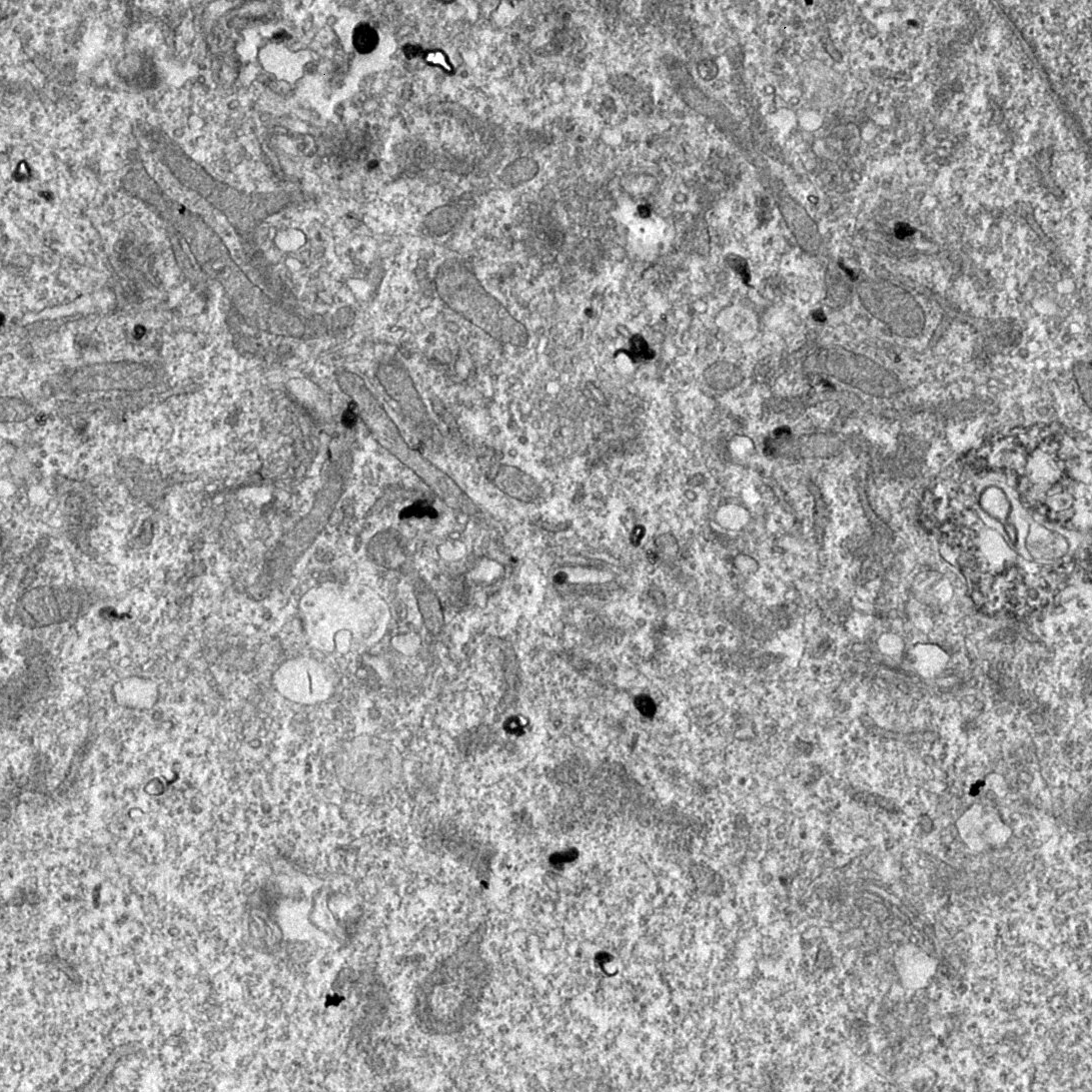

Supplement: Supplementary file 6 [file DataSheet6.ZIP › TEM_final/hFOB_0ug_low magnification.jpg]

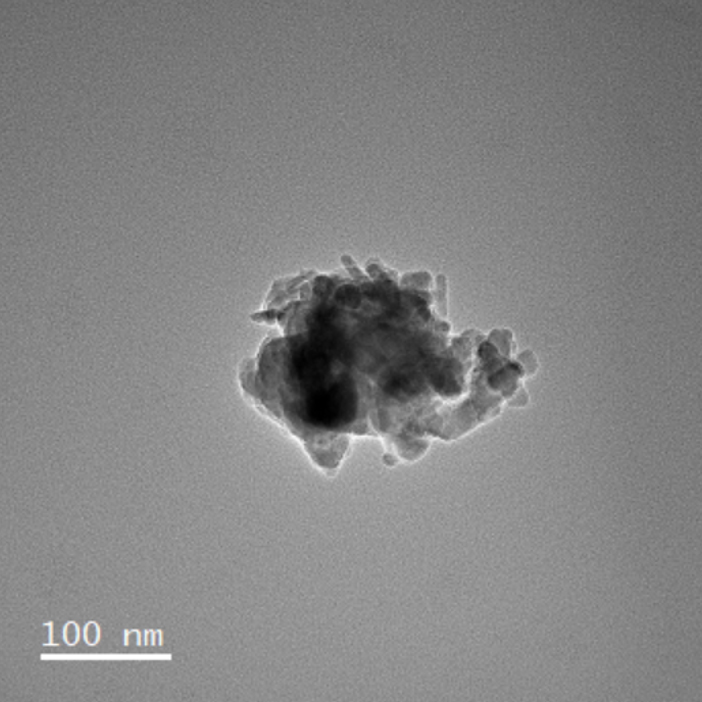

Supplement: Supplementary file 6 [file DataSheet6.ZIP › TEM_final/Nano particle_morphology.jpg]

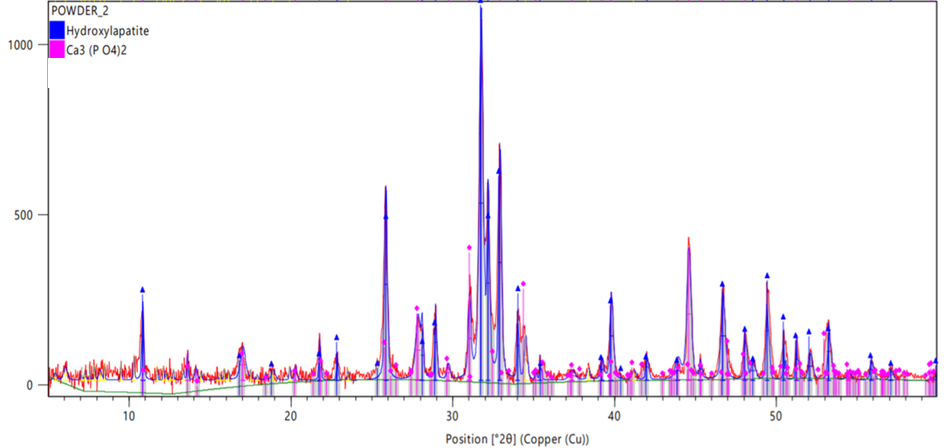

Supplement: Supplementary file 9 [file DataSheet5.ZIP › XDR_final/XRD.jpg]
