# Supplementary material for: Nanoscaled biphasic calcium phosphate modulates osteogenesis and attenuates LPS-induced inflammation
Source: Front Bioeng Biotechnol. 2023 Nov 29;11:1236429. doi: 10.3389/fbioe.2023.1236429 (PMC10716545; doi:10.3389/fbioe.2023.1236429)
Supplement: Supplementary file 7 [file DataSheet1.PDF]

Zeta Potential - Quality 2 Page

Sample Details

**Sample Name:**	bone powder
**Project Name:**	biolab
**Date and Time:**	Tuesday, July 28 2020 12:25:39 PM

**Type:**	Zeta	**Result Source:**	Instrument
**Cell Name:**	DTS1070	**Temperature (°C):**	25
**Material Name:**	Polystyrene latex	**Dispersant Name:**	Water
**Material RI:**	1.59	**Dispersant RI:**	1.33
**Material Absorption:**	0.01	**Dispersant Viscosity (cP):**	0.887
		**Dispersant Dielectric Constant:**	78.5

Zeta Potential Distribution

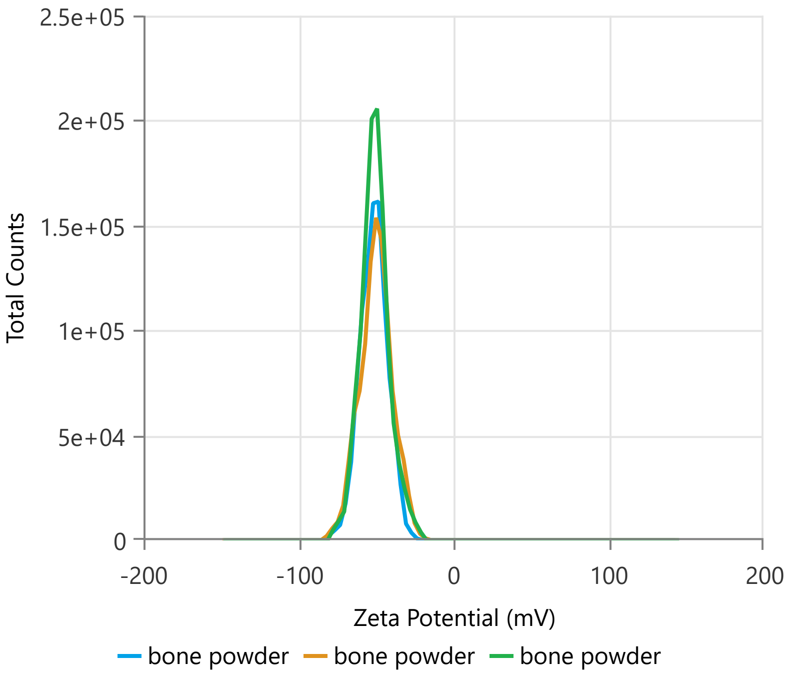

Phase Plot

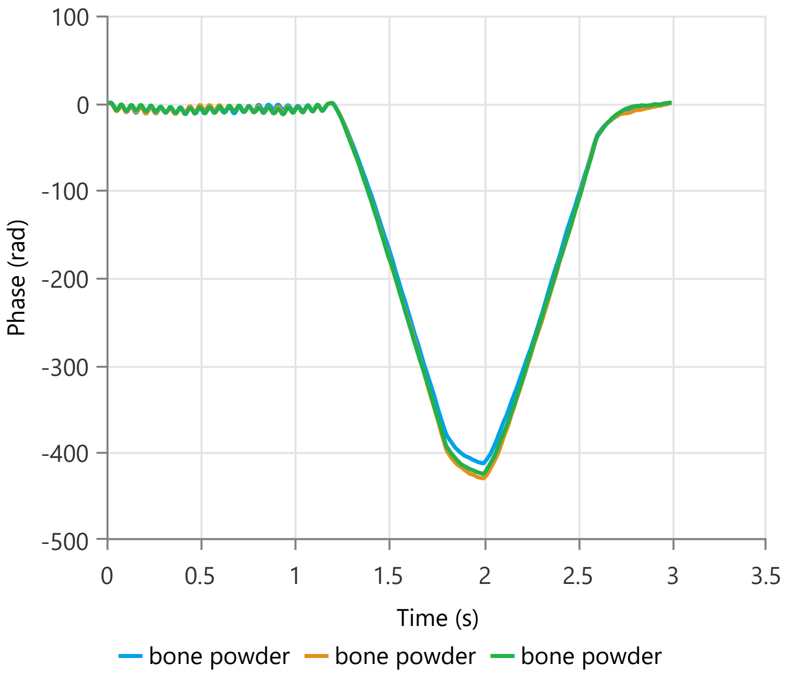

Parameter List

**Number Of Zeta Runs**	: 12
**Zeta Potential (mV)**	: -51.35
**Zeta Deviation (mV)**	: 8.883
**Wall Zeta Potential (mV)**	: -64.31
**Zeta Peak One Mean**	: -50.96
**Zeta Peak One Width**	: 8.785
**Zeta Peak Two Mean**	:
**Zeta Peak Two Width**	:

Parameter List

**Conductivity (mS/cm)**	: 0.9457
**Reference Beam Count Rate (kcps)**	: 2140
**Derived Mean Count Rate (kcps)**	: 2.182E+05
**Mean Count Rate (kcps)**	: 357.9
**Quality Factor**	: 2.543
**Effective Voltage (V)**	: 149.3
**Measured Current (mA)**	: 2.149

Zeta Potential - Quality 2 Page

Zeta Potential Voltage And Current

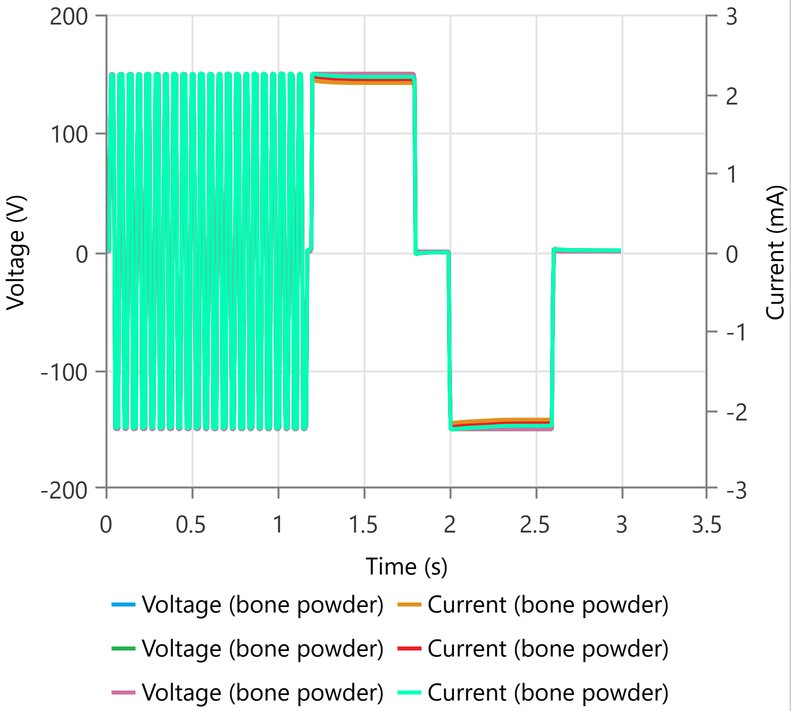

Frequency Shift

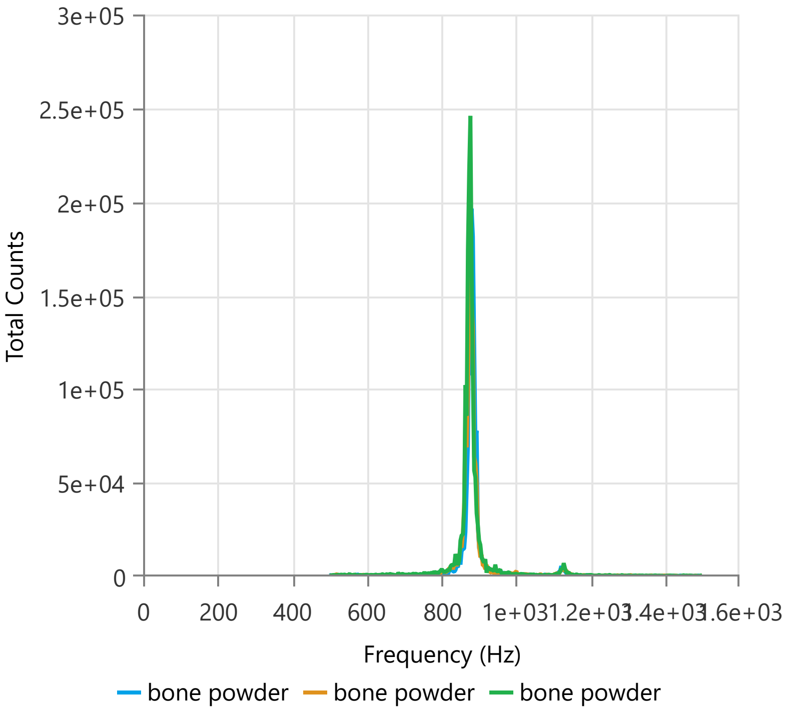

Parameter List

Instrument Serial Number: MAL1233483
